# Associations between rurality and regional differences in sociodemographic factors and the 1918–20 influenza and 2020–21 COVID-19 pandemics in Missouri counties: An ecological study

**DOI:** 10.1371/journal.pone.0290294

**Published:** 2023-08-30

**Authors:** Lisa Sattenspiel, Carolyn Orbann, Aaron Bogan, Hailey Ramirez, Sean Pirrone, Sushma Dahal, Jane A. McElroy, Christopher K. Wikle

**Affiliations:** 1 Department of Anthropology, University of Missouri, Columbia, MO, United States of America; 2 Department of Health Sciences, University of Missouri, Columbia, MO, United States of America; 3 Department of Health Sciences Research, Division of Biostatistics, Mayo Clinic, Scottsdale, AZ, United States of America; 4 Bond Life Science Center, University of Missouri, Columbia, MO, United States of America; 5 School of Medicine, University of Missouri, Columbia, MO, United States of America; 6 School of Public Health, Georgia State University, Atlanta, GA, United States of America; 7 Department of Family and Community Medicine, University of Missouri, Columbia, MO, United States of America; 8 Department of Statistics, University of Missouri, Columbia, MO, United States of America; Drexel University, UNITED STATES

## Abstract

This study compares pandemic experiences of Missouri’s 115 counties based on rurality and sociodemographic characteristics during the 1918–20 influenza and 2020–21 COVID-19 pandemics. The state’s counties and overall population distribution have remained relatively stable over the last century, which enables identification of long-lasting pandemic attributes. Sociodemographic data available at the county level for both time periods were taken from U.S. census data and used to create clusters of similar counties. Counties were also grouped by rural status (RSU), including fully (100%) rural, semirural (1–49% living in urban areas), and urban (>50% of the population living in urban areas). Deaths from 1918 through 1920 were collated from the Missouri Digital Heritage database and COVID-19 cases and deaths were downloaded from the Missouri COVID-19 dashboard. Results from sociodemographic analyses indicate that, during both time periods, average farm value, proportion White, and literacy were the most important determinants of sociodemographic clusters. Furthermore, the Urban/Central and Southeastern regions experienced higher mortality during both pandemics than did the North and South. Analyses comparing county groups by rurality indicated that throughout the 1918–20 influenza pandemic, urban counties had the highest and rural had the lowest mortality rates. Early in the 2020–21 COVID-19 pandemic, urban counties saw the most extensive epidemic spread and highest mortality, but as the epidemic progressed, cumulative mortality became highest in semirural counties. Additional results highlight the greater effects both pandemics had on county groups with lower rates of education and a lower proportion of Whites in the population. This was especially true for the far southeastern counties of Missouri (“the Bootheel”) during the COVID-19 pandemic. These results indicate that rural-urban and socioeconomic differences in health outcomes are long-standing problems that continue to be of significant importance, even though the overall quality of health care is substantially better in the 21^st^ century.

## Introduction

The 1918–20 influenza pandemic was one of the deadliest pandemics in history. Approximately 100 years later, the COVID-19 pandemic is now seen in a similar light. While the pandemics were caused by different viruses, the spread of these two diseases is influenced by similar sociodemographic phenomena. An increasing proportion of the world’s population lives in large cities, many of which continue to undergo significant outbreaks of COVID-19. Consequently, many of the strategies designed to confront COVID-19 have been heavily influenced by the experiences of urban populations rather than those of people who do not live in large cities. For this reason, it is important to carefully consider how the characteristics of rural areas differ from those of urban areas, particularly with regards to known risk factors for the spread of infectious diseases, such as population density, size and composition of households, access to health care resources, socioeconomic status, cultural and ethnic differences, and movement patterns. Additionally, it is important to remember that neither urban nor rural populations exist independently of one another and that contact patterns via geographic proximity and social relationships influence the spread of disease between urban and rural populations in ways that may be visible in mortality or case data.

The study presented here discusses the results of analyses of mortality data from the 1918–20 influenza pandemic and the first two years of the 2020–23 COVID-19 pandemic in the U.S. State of Missouri, as well as morbidity data from the COVID-19 pandemic. All pneumonia and influenza (P&I) deaths occurring in Missouri between January 1, 1918 and December 31, 1920 are included in the analysis of the 1918–20 influenza pandemic. Analyses of the COVID-19 pandemic include Missouri deaths and cases reported between March 7, 2020 and March 9, 2022. Cases and deaths from COVID-19 are still occurring and of concern, but our data consider only the first two years of this pandemic. Thus, in the following we refer to the pandemic we are studying as the 2020–21 COVID-19 pandemic.

The focus of the research presented here is the different pandemic experiences both of urban and rural populations and across different geographic regions within the state. The state’s population distribution has been relatively stable over the last century and 72% of the counties are still classified as rural or semirural using U.S. Census guidelines [1]. These features make Missouri an ideal location to use in urban-rural comparisons of the impact of the 1918–20 influenza and 2020–21 COVID-19 pandemics.

Although numerous studies have considered the spread of the 1918–20 influenza pandemic or the 2020–21 COVID-19 pandemic, research directly comparing the population-level outcomes of both pandemics is scarce. The majority of such papers qualitatively compare biological, epidemiological, and/or behavioral risk factors of importance in the two pandemics, but do not compare mortality or morbidity data [e.g., 2, 3, 4, 5, 6, 7, 8, 9]. Most studies that do analyze epidemiological data from both pandemics are focused on urban populations. For example, Faust et al. [10] looked at all-cause mortality in New York City during both pandemics and found similarities in death rate increases due to the pandemics. They also found lower all-cause mortality in the 21^st^ century in general compared to the years preceding the 1918–20 influenza pandemic. He et al. [11] compared timing of morbidity and mortality waves in the UK during the 1918–20 influenza and COVID-19 pandemics. They found similar wave patterns that they attributed to timing of control measures but did not explicitly address sociodemographic attributes of their population.

The experiences of rural communities in the face of any major outbreak of infectious diseases can be very different from those of urban communities, and many studies have addressed these differences, especially in light of the spread of the COVID-19 pandemic. Most of these studies focus on large-scale geography, such as the entire US or a large region [e.g., 12, 13, 14]; some focus just on urban-rural differences and do not directly consider data on the disease itself [e.g., 15, 16]. Huang et al. [17] did limit their geographic scale, to the U.S. state of South Carolina, and analyzed COVID-19 death data with reference to various risk factors of particular importance in rural communities. Their study was preliminary, however, and was based only on mortality data from the first six months of the pandemic.

Research comparing the spread of both pandemics in Missouri has not been uncovered, and only a limited number of studies of either the 1918–20 influenza or the COVID-19 pandemic in the state exist in the literature. Some studies of the 1918–20 influenza pandemic in Missouri are historical discussions focused on particular cities, and/or do not present detailed epidemiological data on the pandemic within the state [e.g., 18–20]. Garrett [21] considered the pandemic throughout the U.S., but included substantial information on its impact in Missouri. However, all his analyses were based on aggregate data published by the U.S. Census Bureau rather than the individual death certificates used in the present study. Two studies have made use of the Missouri Digital Heritage Death Certificate database [22] to analyze death data quantitatively. Hoffman [23] focused on the timing and spread of the pandemic in St. Joseph, Missouri, while Hoffman and Fox [24] analyzed data for the entire state. The latter study compared total mortality statistics from 1918 through 1923 with a baseline from 1915 through 1917 and found that high numbers of excess deaths were found near cities, in Missouri’s southeastern corner (“the Bootheel”), and in the mining belt in southwestern Missouri and southeastern Kansas. Hoffman and Fox also found that there was widespread negative mortality after major pandemic waves and suggested this provided evidence of harvesting, a situation where mortality declines after a major event because of earlier than expected deaths of those who are most vulnerable.

Studies that consider the spread of the COVID-19 pandemic in Missouri are also rare. McKinsey et al. [25] provide a qualitative overview of the pandemic and focus on the strategies that were implemented to deal with the pandemic at the state level and the political issues and ramifications of those strategies. Several other papers have centered on assessing the degree of geographic disparities with regards to COVID-19 risk and have tried to link these disparities to the geographic distribution of cases and/or deaths. Studies of this type have been carried out on the two major urban areas in the state, St. Louis [26, 27] and Kansas City [28, 29], but no studies dealing with other parts of the state have been identified.

None of the papers focusing on either the 1918–20 influenza pandemic or the COVID-19 pandemic have addressed similarities and differences in the geographic distribution of both pandemics among the different parts of the state. The study presented here fills that gap. It clusters Missouri’s counties using similarities and differences in a set of sociodemographic characteristics for which data are available for both time periods. A second clustering of counties is made using rural/semirural/urban status. Cumulative mortality and morbidity rates are compared among the sociodemographic and rural/urban clusters and excess all-cause mortality rates are derived for the two pandemics at the state level. The overall purpose of the study is to provide insight into how rural/urban status and different sociodemographic factors that can be measured by data collected routinely at a county-level may be associated with patterns of disease transmission and severity, in hopes of developing better planning and implementation of public health measures for future disease outbreaks.

The study adds to the existing literature by emphasizing explicitly the different pandemic experiences of urban and rural populations in a U.S. state that has not been well studied. It uses a comparative framework that draws on both the 1918–20 influenza pandemic and the 2020–21 COVID-19 pandemic. The comparison is not perfect since the pandemics were 100 years apart and the conditions under which pandemics spread have changed in important ways. As both our research and a study of London’s social environment across 100 years [30] have shown, however, many aspects of the environmental contexts within which pandemics spread are remarkably stable across long periods of time. A comparison such as that described here provides a time depth needed to determine which characteristics of a region may be stable over the long term and which are more short-lived. This can enable better evaluation of the importance of different risk behaviors and situations in determining the potential for major epidemic experiences and increases insight into whether and how such situations may be tackled to lessen future pandemic risks.

## Characteristics of Missouri during the two pandemics

A major influence on the spread of any pandemic is the social and environmental context within which the disease is spreading. This research focuses on a single state in the United States, but on pandemics that were a century apart. There are both similarities and differences in the context surrounding those pandemics. Here we describe some of the major elements of the context in the early 20^th^ and 21^st^ centuries that are most likely to have affected the risks and nature of pandemic spread, including the demographic structure of the population, the nature of transportation, and the types and availability of healthcare.

Demographic variables such as total population, sex ratio, proportion living in urban and rural communities, ethnic diversity, and types of employment strongly influence general levels of individual and community-level transmission risks and the potential severity if infected. Infectious diseases such as influenza or COVID-19 generally spread rapidly in crowded conditions of high population density. The nature of transportation influences the potential rates and patterns of contact among regions, which directly affects spread of a pandemic across the landscape, and the basic nature of health care available to the general population of Missouri at each time period had direct consequences for individual health.

We discuss first the Missouri of 2020, as this will be most familiar to present-day readers. We follow this with a description of the social and environmental context prevalent in the Missouri of the early 20^th^ century, and end with a brief comparison of the two time periods, focusing on features we think are most important in understanding the nature of the two pandemics under consideration.

### Missouri in the 21^st^ century

Missouri is the 21^st^ largest state in area in the U.S. and consists of 114 counties and St. Louis City (hereafter included as one of 115 counties). It lies in the middle of the United States, close to the geographic center of the continental U.S. and containing the mean center of the present U.S. population [31]. The state has two major Metropolitan Statistical Areas (MSAs)–St. Louis on the east central border and Kansas City on the west central border, along with two other MSAs with populations over 200,000, Springfield in the south-central and Columbia in the central region of the state [32]. It also has three other sizable MSAs—Joplin in the southwestern corner of the state, Jefferson City in the central region, and St. Joseph along the northwestern border [32]. Outside of these places the state is largely rural. Except for Columbia and Jefferson City in the central part of the state, all of these population centers were evident in the early part of the 20^th^ century, when the influenza pandemic of 1918–20 spread through American communities.

The U.S. Census Bureau defines an urban county as one in which 50% or more of the county’s population lives in either “urbanized areas”, incorporated places of 50,000 or more people, or “urban clusters,” incorporated places adjacent to urbanized areas and with a population between 2500 and 50,000 [1]. Using this definition, 32 of the 115 counties in Missouri are currently classified as urban, while 83 are rural. About 1/3 of the total population currently lives in rural areas [33]. All counties that were urban in 1918 are still urban, and the majority of counties that were rural in 1918 are still classified as rural in 2020 by the U.S. Census Bureau (S1 Table). In addition, in the 100 years between the pandemics, there has been no change in the number or boundaries of the state’s counties.

The 2020 Missouri population was 6,154,913 persons, which ranks it 19th in the U.S.; females make up 50.6% of the population [34]. Over half of Missouri residents live in the St. Louis or Kansas City MSAs. In comparison to the U.S. as a whole, Missouri’s rate of population growth is lower (0.2% vs. 0.4%) and the percentage of high school graduates is higher (91% vs. 88.9%), but the percentage with at least a bachelor’s degree is lower (30.7% vs. 33.7%). The state also has substantially less racial and ethnic (especially Hispanic) diversity (78.7% vs. 59.3% White, non-Hispanic; 4.7% vs. 18.9% Hispanic), a lower percentage of foreign-born (4.2% vs. 13.6%), a smaller household size on average (2.5 vs. 2.6 persons per household), and lower median household income ($61,043 vs. $69,021). The age-sex distribution is similar to that of the rest of the U.S. [34]. The primary industries in the state at the present time are health care and social assistance, retail trade, and manufacturing [35].

As is true for most of the U.S., transportation occurs primarily by personal car. Missouri is bisected by two major east-west interstates (I-70 and I-44) and one north-south interstate (I-35). Passenger trains, which carry only a small number of passengers, run east-west between St. Louis and Kansas City and southwest to northeast from Kansas City to Chicago. No significant human travel occurs by water along the Missouri or Mississippi Rivers.

The distribution and nature of health care at the present time is quite different from that present in the early 20^th^ century. Between 10% and 20% or more of working age residents in almost all Missouri counties are uninsured [36]. A significant percentage of the rural counties do not have affordable health clinics, and approximately 11% of Missouri’s total population live in a county with no hospital; for these persons the closest hospital is 14–52 miles away (average 29 miles) [37]. Missouri also has one of the lowest percentages of health care needs met in the entire country [38]. Access to care varies considerably across the state, however. As a consequence, health screenings and preventive care as well as obtaining timely needed medical care for trauma, serious illnesses, and chronic conditions vary dramatically among the counties.

### Missouri during the 1918–20 influenza pandemic

The information used to describe the demographic structure of Missouri in 1918 was collected from the 1910 Missouri census as this document is the closest census prior to the pandemic. The U.S. Census Bureau only produces decennial censuses for each state and despite an exhaustive search, information could not be found on sociodemographic characteristics of the state during the intercensal years. The 1920 Census was closer in time to the pandemic, but it was not chosen to represent the pandemic time period as it reflects major sociodemographic changes caused by the 1918–20 influenza pandemic itself and World War I. A comparison of data from the 1910 and 1920 censuses indicates that, although there were changes during the decade, they were moderate in scope. For example, the overall population increase between 1910 and 1920 was 110,720 people (3.4%), but this was half as large as the increase between 1900 and 1910, a time period that was not influenced by major events such as WWI and a global influenza pandemic that were present in the later decade [39]. Cities were also increasing in size at the expense of rural areas, but as was the case for the total population, these changes were relatively slow between 1910 and 1920 and the rate was lower than what was observed between 1900 and 1910. These and other changes indicate that the sociodemographic characteristics measured in the 1910 census, although not a perfect reflection of those characteristics in 1918, are a reasonable proxy for 1918.

In 1910, Missouri was the 7^th^ most populated U.S. state, with a total of 3,293,335 inhabitants. The sex distribution in 1910 was slightly different from the 2020 population—48.75% of the population was female [40]. The county divisions were the same in 1910 as they are at present, although the census did not designate counties as urban or rural. That designation was reserved for communities, which were deemed to be urban if they were incorporated and had over 2500 inhabitants. The census did indicate the percentage of state and county residents that lived in communities designated as urban, however. Over half (57.5%) of the population lived in communities with fewer than 2,500 inhabitants or in other smaller rural landscapes, making the state substantially more rural in 1910 than in 2020.

The state was less racially diverse in 1910 than in 2020–95% of the population identified as White (native and foreign-born) [40]. Black individuals constituted 4.8% of the population, and the remaining 0.2% of the population’s makeup consisted of Chinese, Japanese, Indian, and other ethnicities. The majority of Missouri’s non-White population lived in urban areas, a pattern that is still true today, although a substantial minority (about 1/3) lived in rural areas.

In 1918, agricultural employment was most common; 78.6% of Missouri land consisted of farmland [40, 41]. The manufacturing industry and trade employment were the next most common, likely due to those occupations’ support of the agricultural industry [41]. Mining was also an important source of employment, especially in the Southern parts of the state [42]. While agricultural employment often was relatively solitary, manufacturing and mining usually involved enclosed workspaces, which would facilitate the spread of diseases such as influenza.

Ownership of automobiles increased dramatically during the years before the 1918–20 influenza pandemic [43], but the main mode of transportation was by railroad. Railroads in Missouri were not used extensively by the public during 1917–1920, however, as they were appropriated by the federal government for World War I efforts [44].

In early 20^th^ century Missouri, it was already understood that urban and rural communities faced different sets of challenges and levels of health disparities [45]. Both hospital beds and physicians were disproportionately clustered in higher population counties [46, 47]. Thus, some aspects of the heterogeneity observed today were present in 1918.

It is more difficult to assess other domains by which health care is usually considered. Health insurance was not widely available in 1918, and most health spending was out of pocket. Charity homes could provide care to specific populations (e.g., unwed mothers or the elderly), but these were privately run, and little information is available about them. Additional health care resources included state-run inpatient facilities for high-need populations, such as epileptics, people with severe mental health needs, or people with tuberculosis [48].

Health care quality may be even more difficult to assess than capacity. According to early 20^th^ century State Board of Health reports, the primary concern was how best to implement sanitary and hygienic measures [45]. Control of infectious disease was the main priority, but available tools still focused on non-pharmaceutical measures like cleanliness and food and water safety. Although the state had a laboratory used for pathogen identification, it was not able to provide timely diagnoses of viral diseases, such as influenza. So, while there were advances at the state laboratory in evidence-based protocols for clinical diagnosis, treatment, and control of many infectious diseases, implementing these advances in treating patients at point of care was limited.

This comparison illustrates that many aspects of life in Missouri in 1918 were similar to those in 2020, but there were also significant differences. Similarities include the high proportion of rural land, the geographic locations of urban centers, and relative homogeneity of the population across counties in terms of the age, sex, and racial and ethnic distribution. Differences include better roads and nearly universal access to vehicles, resulting in greatly increased mobility in the 21^st^ century. Although the number and locations of urban areas are similar, their resident populations are much greater today, increasing the risks of person-to-person transmission of infectious diseases. The predominant occupations have changed significantly, with marked declines in mining and agricultural activities and increases in health professions, the retail sector, and manufacturing. These changes likely have had effects on underlying risk factors for a variety of health conditions. Access to health care during both time periods was heterogeneous, with substantially greater availability and access in urban counties than in rural counties. Treatment of infectious diseases in 1918 was much less sophisticated than at present. This suggests that the outcomes for those who were afflicted with influenza in 1918 may have been more similar throughout the state due to fairly equal treatment regimens, while outcomes in 2020 may exhibit more obvious differences between residents of rural vs. urban counties due to variability in accessing appropriate treatments. In light of these comparisons across the last century, we turn to the two pandemics bounding that century and discuss their impacts overall, across different regions of the state, and in urban vs. semirural vs. rural counties.

## Materials and methods

This project was reviewed by the MU Institutional Review Board, who determined that it did not constitute human subjects research according to the U.S. Department of Health and Human Services regulatory definitions.

We address two aspects of pandemic spread during both time periods: a) rural/semirural/urban (indicated as RSU) differences, and b) differences among regions defined by sociodemographic characteristics. Data available include mortality data from both the 1918–20 influenza and the 2020–21 COVID-19 pandemics, morbidity data from the 2020–21 COVID-19 pandemic, and sociodemographic data from the U.S. census, government organizations, and other archival sources.

### Selection of sociodemographic variables

Data on 20–30 different sociodemographic variables were initially collected for each time period. To ensure comparability of analyses of both pandemics, however, data used in the study were limited to a smaller set of variables that adequately measured similar population characteristics.

The variables used in analyses by county included total population size, proportion male, ratio of people aged 20–44 to those aged 65 and older, proportion White, literacy rate in 1910 and high school graduation rate in 2020, average value of a farm, and number of primary care physicians per 100,000 persons. Proportion Hispanic was included in analyses of the 2020–21 COVID-19 pandemic, but in 1910 Hispanic proportions were very low and data were unreliable, so the variable was omitted from analyses of the influenza pandemic. Variables chosen for the analyses were minimally correlated with one another. The values used for the chosen variables are provided in S1 File.

Several challenges arose during data collection, especially for data from the early 20^th^ century. For example, the 1910 Missouri census had to be used to describe the demographic structure of Missouri in 1918 as this document is the closest census prior to the pandemic. Sources other than the 1910 Missouri census, with state-level information on income, education, or living standards could not be identified.

County-level data were especially sparse for the early 20^th^ century. Population numbers were provided for some age categories, but at the county level these were not the standard 5-year age categories used in demographic data today and these sometimes changed from census to census.

It is probable that the 1910 population and death counts for non-White residents were underreported as they did not have the same rights or treatment as White individuals. In addition, regulations requiring death registration were just becoming widespread at the time of the pandemic—Missouri was first included in the U.S. registration area in 1911 [49], so deaths in all age groups, and especially young children, were likely under-recorded. This problem was probably worse for underrepresented groups [50, 51].

*Polk’s Medical Register* [47] was used to provide data on health care access during the early 20^th^ century. These data are imperfect estimates, as all types of health care providers were conflated into the umbrella terms of physician or surgeon. No documents describing household-level economic variables, such as per capita income by county were available; the only economic information of that type uncovered was the average value of all farm property per county provided by the 1910 Missouri Census Supplement [40].

The opposite problem occurred with the collection of sociodemographic data during the 2020–21 COVID-19 pandemic—there exists an overabundance of all types of data. The goal of identifying a common set of variables from both time periods as well as minimizing the correlations among variables motivated the final choice of variables used in the analyses.

### Using sociodemographic data to aggregate Missouri’s counties

To enable the comparisons of the influenza and COVID-19 pandemics, two methods were used to aggregate the state’s counties: a) distribution based on the urban and rural proportions in their respective populations, and b) sociodemographic similarity (S1 Table). The criteria used for both classification schemes did not include any specific disease-related input and thus the classifications provide independent foundations to use in evaluating the spread of each virus—influenza or SARS-CoV-2.

The RSU (rural/semirural/urban) classifications for both 1918 and 2020 were based on the urban proportion of a county’s population reported in the 1910 and 2010 censuses and the present definition of “urban” used by the U.S. Census Bureau (a county with over 50% of the population living in urbanized areas or urban clusters) [1]. The 2010 census was used because criteria for determining the rural/urban classifications for the 2020 census were being revised and the new designations were not available at the time the analyses in this paper were completed. Within the rural category we have defined a subgroup, semirural, to include those counties in which over 50% but less than 100% of the residents live outside of urbanized areas or urban clusters. Those counties with a totally rural population are classified as “rural”.

County-level sociodemographic variables were used to group counties into four distinct regions in both 1918 and 2020. In each time period, Ward clustering was performed on these variables using the ClustGeo package in R [52]. This method assigns cluster membership by minimizing the Euclidean distance between county sociodemographic variables and results in clusters of counties that are sociodemographically similar. To account for geographic similarities, county centroids were used to introduce a weighted contiguity constraint. The constraint provided a means of selecting the amount of influence physical proximity had on the sociodemographic homogeneity of each cluster. Selecting a reasonable weighting of the physical space dimension relative to the feature space was accomplished by choosing a value that minimized the reduction in sociodemographic similarity while maximizing the influence of spatial proximity. Using a Ward dendrogram of all possible cluster divisions based on sociodemographic features as well as input from project collaborators, four clusters were selected for both time periods.

Once the clusters were defined, a random forest algorithm [53] was used to assess the relative importance of each individual sociodemographic variable for determining group/cluster membership. The goal in evaluating individual variable importance was to provide additional interpretability when assessing differences in disease progression between the four county clusters. Descriptive labels were used to characterize each of the regions for a particular time period, but it is important to note that the clustering procedures were done independently on the variables from each time period, with the result that the county composition of each region, although similar, is not identical for the two periods.

### Disease data from the 1918–20 influenza pandemic

The primary source of data on influenza mortality used in this project is the Missouri Digital Heritage death certificate database [22], which contains all death certificates held by the state and filed between 1910 and 50 years before the present (a rolling boundary that changes each year). Birth date, death date, age, sex, and other demographic data were collected on all people who died from pneumonia and influenza in all 115 Missouri counties during 1918, 1919, and 1920. Missouri became a death registration state and reported mortality statistics to the U.S. Census Bureau beginning in 1911. Thus, the state is listed in the U.S. Mortality Statistics reports [e.g., 49] after this time. Data in these reports provide information only at the state level and occasionally for selected cities, however, so data culled from death certificates, which provide death statistics at the county level, have been used for most analyses in this paper.

Additional information on the 1918–20 influenza pandemic was collected from newspaper reports, State Board of Health Biannual reports, and other ephemera found in archives. These information sources provide illustrative details about control measures and the social climate that existed in Missouri during the pandemic period, but rarely provide comprehensive quantitative data on epidemic outcomes.

### Disease data on the 2020–21 COVID-19 pandemic

Both county-level case and mortality data dated between March 7, 2020 and March 9, 2022 were obtained directly from the Missouri Department of Health and Senior Services (MODHSS) or by using their public facing dashboard. The data capture the first 4 waves of the pandemic (through the first omicron wave). Subsequent to this time, reliable comprehensive reporting and publication of data was affected by the increasingly greater availability of vaccination and self-testing and the waning of the first omicron wave.

### Analysis of the mortality and morbidity data

Results are presented below for morbidity (COVID-19 only) and mortality patterns (both pandemics) by RSU status and sociodemographic region. Because some counties had low numbers of deaths, especially at the time of the influenza pandemic, all analyses of death data aggregated the death counts according to the groups defined by RSU status or the sociodemographic cluster analysis. Data were also not available for age-adjustment during the 1918–20 pandemic, so to maintain comparability of both pandemics, crude mortality rates were calculated (referred to as mortality rates in subsequent sections). Cumulative mortality and morbidity patterns were calculated directly from the relevant daily death or case data. Counties were grouped as previously described, and cumulative deaths per 100,000 persons in these aggregated regions was used to compare regional mortality. Time series of deaths/100,000 persons or cases/100,000 persons were used to visually compare the county groupings.

### Estimation of excess all-cause mortality

The mortality patterns associated with both the 1918–20 influenza and 2020–21 COVID-19 pandemics in the state were quantified by estimating monthly all-cause excess mortality rates per 10,000 population. County-level monthly mortality figures are not easily accessible for either pandemic; thus, excess all-cause mortality estimates were calculated only for the state as a whole. For the influenza pandemic, total monthly deaths for 1911–21 were extracted from the Missouri Digital Heritage database [22]. Annual population figures were published in a report from the U.S. Bureau of the Census [54]. For the COVID-19 pandemic, monthly all-cause death totals for 2011–21 were reported in the Monthly Vital Statistics reports of the Missouri Department of Health and Senior Services [55]. Population estimates for those years were derived from U.S. Census data and were downloaded from https://www.macrotrends.net/states/missouri/population. Complete COVID-19 mortality data were not available for 2022, so excess all-cause mortality during the COVID-19 pandemic was determined only through the end of 2021. All-cause excess mortality rates were found by computing the mortality rate above a seasonal baseline of expected mortality rates in the absence of influenza or COVID-19 activity.

The time series of monthly all-cause deaths during each pandemic was first examined to estimate the timing of pandemic influenza or COVID-19 activity. The months that were determined to be associated with pandemic activity were excluded so that a baseline non-pandemic mortality rate could be modelled. The baselines for the two pandemics and their 95% confidence intervals were estimated by fitting cyclical Serfling regression models to all-cause deaths for the total populations in non-pandemic months, with separate models used for each pandemic, as done in previous studies [56–59]. Temporal trends and harmonic terms for seasonality were included in these linear regression models [60]. Periods of pandemic activity were then defined for influenza as the months in 1918 through 1920 where the observed total all-cause mortality rate per 10,000 population was greater than the upper 95% CI of the baseline. The same strategy was used to determine periods of COVID-19 pandemic activity.

For both pandemics, the excess mortality rate was defined as the difference between the observed and model-adjusted baseline mortality rates for each month within a pandemic wave. The excess death rates were summed across the pandemic months from 1918 through 1920 to get the overall pandemic excess mortality during the influenza pandemic; excess death rates were summed across the pandemic months in 2020 and 2021 for the COVID-19 pandemic, in line with previous studies [56–59]. All excess mortality analyses were completed using SAS 9.4.

## Results

### The rural/semirural/urban (RSU) classification and sociodemographic clustering of the state’s counties

The proportion of residents living in rural areas in most Missouri counties decreased with economic development and population growth between 1910 and 2010. The RSU groups remained stable for about half of the counties (n = 61) and all urban counties in 1910 were still classified as urban in 2010. The remaining changes in RSU classification were as follows: semirural in 1910 to urban in 2010 (n = 18), rural in 1910 to semirural in 2010 (n = 30), and rural in 1910 to urban in 2010 (n = 6) (S1 Table).

**Fig 1:**
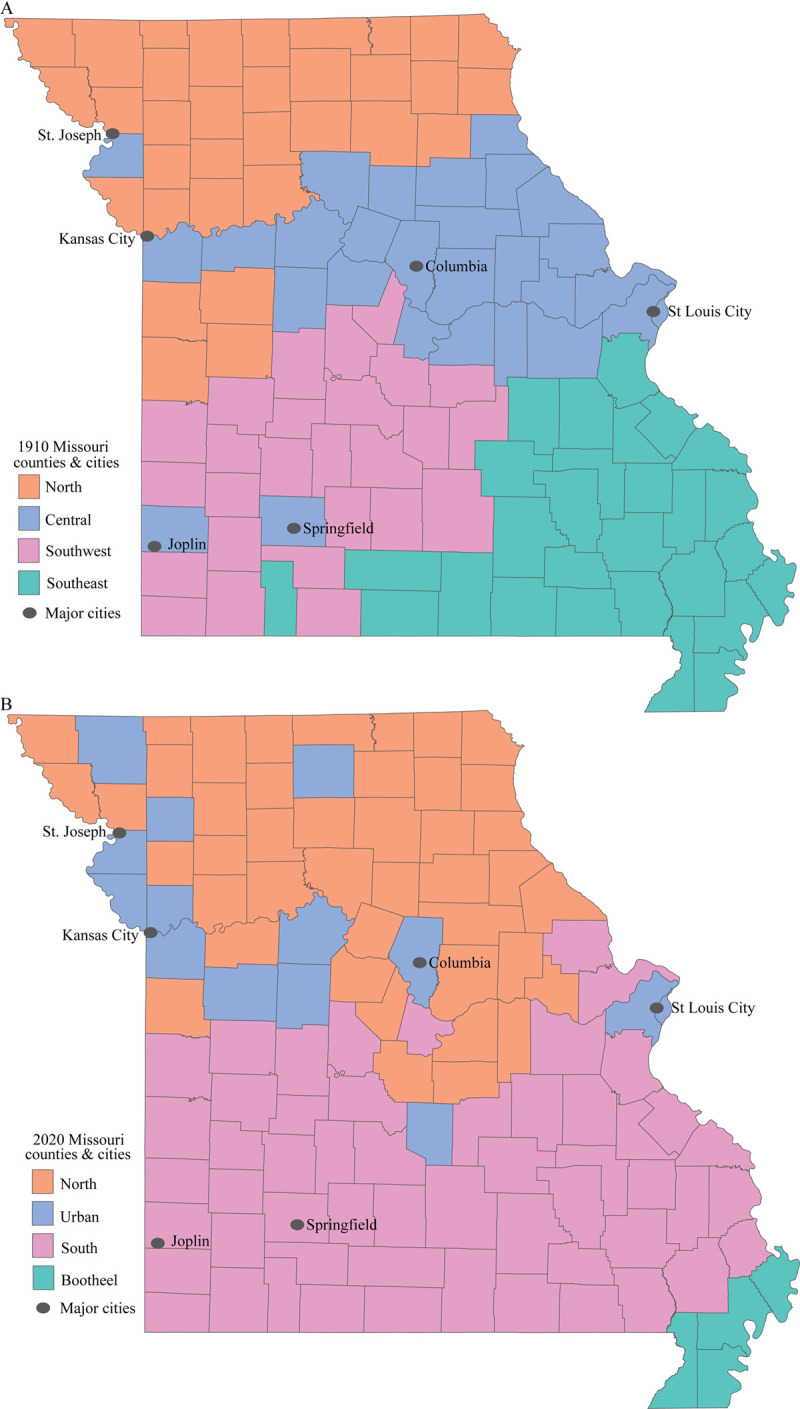
Classification of Missouri counties into four regions based on sociodemographic characteristics. (A) The 1910 classification. (B) The 2020 classification. Source: Authors’ original figure. Data for the base map is in the public domain and available from the State of Missouri Data Portal at https://data.mo.gov/Demographics/Missouri-County-Boundaries-Map/n34b-fwqr. The data made available here has been modified for use from its original source, which is the State of Missouri. THE STATE OF MISSOURI MAKES NO REPRESENTATIONS OR WARRANTY AS TO THE COMPLETENESS, ACCURACY, TIMELINESS, OR CONTENT OF ANY DATA MADE AVAILABLE THROUGH THIS SITE. THE STATE OF MISSOURI EXPRESSLY DISCLAIMS ALL WARRANTIES, WHETHER EXPRESS OR IMPLIED, INCLUDING ANY IMPLIED WARRANTIES OF MERCHANTABILITY, OR FITNESS FOR A PARTICULAR PURPOSE. The data is subject to change as modifications and updates are complete. It is understood that the information contained in the Web feed is being used at one’s own risk.

Fig 1 illustrates the location within the state of the sociodemographic clusters for the two pandemic time periods; the designated cluster for each county is also indicated in S1 Table. In both time periods, the counties were separated into four groups. In 1910 the southern half of the state separated into distinct southeastern and southwestern clusters, while the other two clusters consisted primarily of central and northern counties (Fig 1A). The two counties in the southwestern part of the state that fell into the central group included the cities of Springfield and Joplin (SW portion of the state), both of which were urban counties.

The clustering in the 21^st^ century is broadly similar, but has some distinct differences. The urban grouping in 2020 is mostly limited to the highly urbanized parts of the state and does not consist of geographically contiguous counties (Fig 1B). The counties containing Springfield and Joplin are not included in the urban group in 2020, most likely due to broad similarities in the chosen sociodemographic characteristics across the southern half of the state. The other feature that is distinctive about the 2020 regional grouping is that the Bootheel region in the southeastern corner of the state forms its own sociodemographic cluster. This region is known to be socially, culturally, and economically distinct from much of the state [61], and it is significant that the variables we chose capture the uniqueness of the region.

For both time periods, the random forest algorithm identified average farm value, proportion White, and literacy as the most significant sociodemographic variables that determined cluster membership. Two additional variables, proportion Hispanic and age ratio had an appreciable influence on the 2020 county clusters as well.

### General pattern of the 1918–20 influenza pandemic in the State of Missouri

The pattern of spread of the global 1918–20 influenza pandemic has been characterized as a series of three waves occurring during an approximate one-year period in 1918 and 1919 [62, 63]. A growing body of recent research suggests that many regions, including the State of Missouri, experienced a substantial fourth wave of the pandemic in 1920 [58, 64–71].

**Fig 2:**
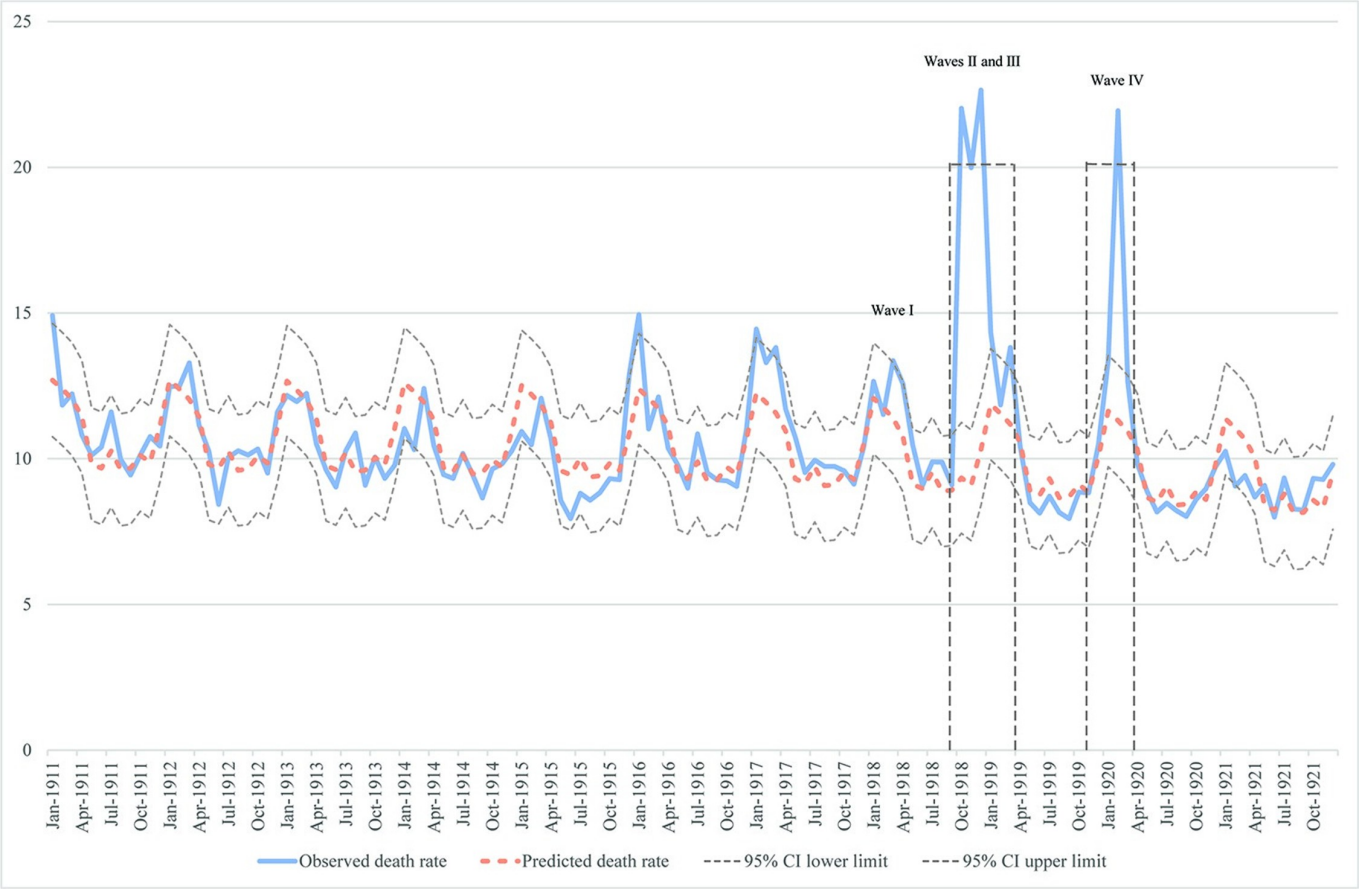
All-cause excess deaths in Missouri during the 1918–20 influenza pandemic. Waves occurred over the following spans: I—early 1918 (through April), II/III—September 1918-April 1919, IV—November 1919-April 1920. Dashed boxes show pandemic periods. Note: During Wave I of the 1918 influenza pandemic there was only a single month with statistically significant excess mortality; thus, this wave is not indicated as a pandemic period.

The first wave in Missouri (Spring 1918) was unremarkable, with an all-cause excess mortality rate of 1.98 (95% CI 0.07, 3.88) per 10,000 population (Figs 2 and S1). By the start of the second wave, it was recognized that the pandemic was more serious than normal and newspapers and public health authorities warned of increased severity, leading to mandatory social distancing and closures of many non-essential businesses in major cities [19]. This fall wave was bimodal with peaks in October and December 1918 and highly significant excess mortality of 12.69 deaths per 10,000 persons (95% CI 10.79, 14.59) in October and 12.34 per 10,000 persons (95% CI 10.44, 14.25) in December. The ending of the wave is unclear due to the presence of a small third wave in the early spring of 1919 (Figs 2 and S1). Thus, our all-cause excess mortality analyses lump waves II and III into the time period from September 1918 to April 1919. In a fourth wave of the pandemic (November 1919-April 1920), an additional and substantial peak in influenza mortality occurred; in fact, this mortality peak was higher than that of the second 1918–19 wave in over 25% of Missouri’s 115 counties. The excess all-cause mortality estimates for the fourth peak were highly significant in February 1920, with a rate of 10.63 deaths per 10,000 persons (95% CI 8.71, 12.54). Overall, the all-cause excess mortality during the 8 months of Wave II/III and 6 months of Wave IV was 51.6 deaths per 10,000 persons (95% CI 40.2, 63.1) and 6 months of that 14-month period exhibited statistically significant excess mortality. This level of excess mortality is substantially higher than that reported by Hoffman and Fox [24], who used a different calculation method, but the pattern of mortality observed in their data [24, Fig 2] is that same as that presented here (Figs 2 and S1).

The timing and severity across time of the pandemic in Missouri was somewhat different from that of the United States as a whole. Missouri’s death rate from pneumonia and influenza (P&I) per 10,000 population was substantially lower in 1918 (47.7 vs. 58.7 deaths per 10,000) and somewhat lower in 1919 (20.6 vs. 22.2 deaths per 100,000 population), but substantially higher in 1920 (26.1 vs. 20.6 deaths per 10,000 population) [72]. These and the excess mortality illustrated in Fig 2 confirm the severe impact of the pandemic in 1920 in comparison to the rest of the U.S. In fact, of all the death registration states reporting in 1921, only Colorado experienced more P&I mortality in 1920 than did Missouri.

### The Missouri COVID-19 Pandemic (Mar 2020-Mar 2022)

**Fig 3:**
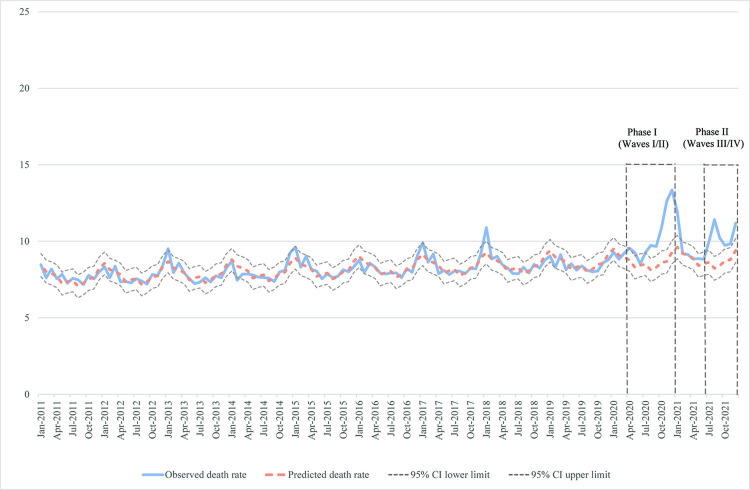
All-cause excess deaths in Missouri during the 2020–21 COVID-19 pandemic. The following waves can be observed: I—April-June 2020, II—July 2020-January 2021, III—July 2021-October 2021, IV—November 2021-end of data collection (shown only through December 2021; see S1 Fig for remaining months). Dashed boxes show pandemic periods.

The first case of COVID-19 was reported in Missouri on March 7, 2020 with the first death reported on March 22, 2020. From this beginning to the end of data collection (March 9, 2022), Missouri experienced four waves of COVID-19, most of which seem to be closely associated with the circulation of new strains of the SARS-CoV-2 virus. During the time period covered by the present study, the state experienced an early, small wave between March and June 2020. This wave peaked in April 2020 with an all-cause excess mortality rate of 0.77 deaths per 10,000 people (95% CI 0.03, 1.52). A large second wave occurred between July 2020 and January 2021; it peaked in December 2020 with all-cause excess mortality of 4.06 deaths per 10,000 people (95% CI 3.31, 4.82). Missouri experienced a brief respite from the pandemic between February and June 2021, but from July through October 2021 there was a third wave. Deaths in this wave peaked in mid to late August 2021 (all-cause excess mortality of 3.18 deaths per 10,000 people, 95% CI 2.40, 3.95). A fourth wave occurred between November 2021 and the end of data collection with peak mortality in early February 2022 (Figs 3 and S1). Excess all-cause mortality calculations could not be made for the fourth wave due to a lack of available data, but it was remarkable for much higher case numbers than any of the previous waves, but lower case fatality rates than the second wave.

The time span of the pandemic covered by our data divides naturally into two phases, from April 2020 through January 2021 (Waves I and II) and from July-December 2021 (Waves III and IV). During the 10 months of Phase I, the all-cause excess mortality was 18.0 deaths per 10,000 people (95% CI 11.3–24.8); 9 of these 10 months exhibited statistically significant all-cause excess mortality. The all-cause excess mortality for the 6 months of Phase II was 10.2 deaths per 10,000 people (95% CI 5.5–14.9) and all 6 months exhibited statistically significant all-cause excess mortality. Combining these two phases leads to an overall estimate for the all-cause excess mortality of 28.3 deaths per 10,000 people (95% CI 16.8–39.8).

Subsequent waves with lower case numbers and markedly lower mortality than the fourth wave have occurred since data collection for the present study ended. Interested readers can learn more about Missouri’s COVID pandemic on the CDC COVID Data Tracker.

### RSU comparisons throughout the two pandemics

**Fig 4:**
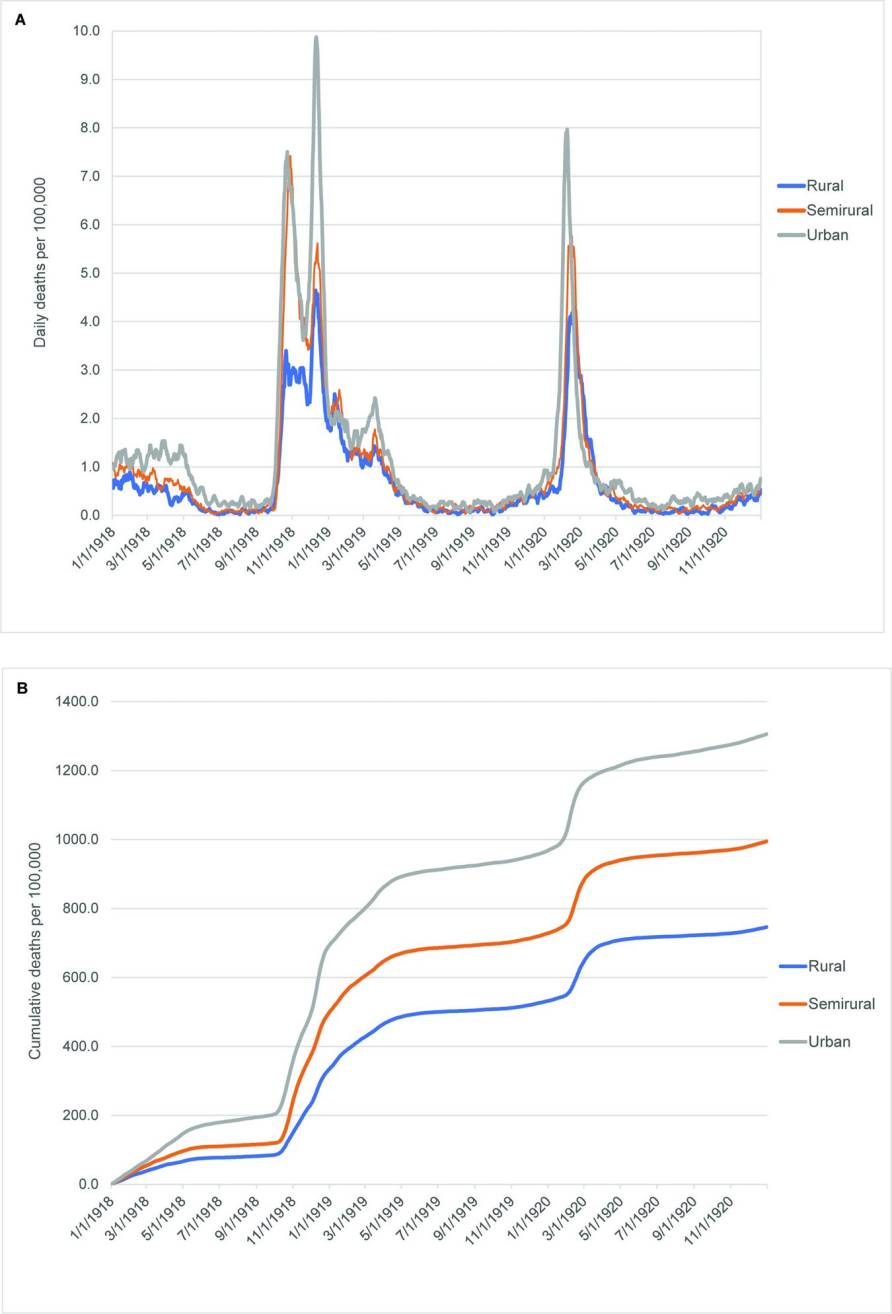
Daily and cumulative influenza mortality curves among groups defined by rural status, 1918–20. (A) Daily influenza deaths per 100,000 people. (B) Cumulative influenza deaths per 100,000 people. All curves are 7-day rolling averages of the data within each county cluster. Pandemic peaks begin when the curves rise most steeply.

Daily and cumulative mortality curves for the RSU groups during the 1918–20 influenza pandemic are illustrated in Fig 4. Fig 4A indicates that the pandemic occurred in a similar pattern in all three regional groupings, with some temporal separation during the 1920 wave. This final epidemic peak occurred first in the urban RSU group, around February 1, followed a week later in the semirural RSU group. The rural RSU group peaked about two weeks after the urban peak. The similar patterning of the pandemic in all regions is also indicated in Fig 4B, which exhibits parallel trajectories in the crude mortality rates per 100,000 persons throughout the 1918–20 pandemic, especially during and after the rapid rise of the second wave in October 1918. The rural RSU group exhibited the lowest cumulative crude mortality rate throughout the pandemic, with the urban RSU group consistently at the highest cumulative crude mortality rate. Fig 4A and 4B also indicate that urban counties experienced substantially more mortality early in the pandemic than did non-urban counties. A high 1918–20 influenza mortality rate in the cities was also noted by Hoffman and Fox [24].

**Fig 5:**
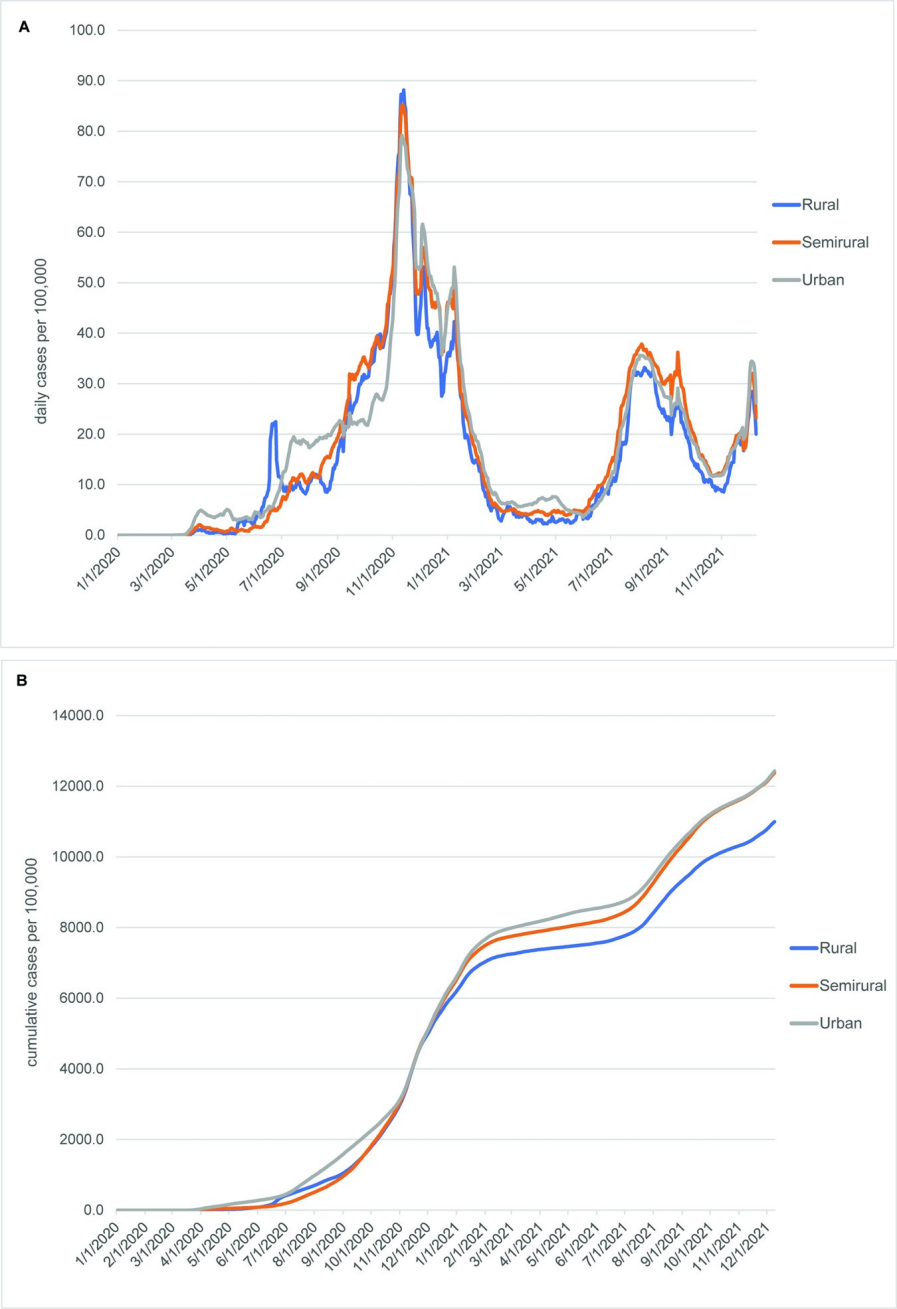
Daily and cumulative COVID-19 case curves among groups defined by rural status, March 2020-December 2021. (A) Daily cases per 100,000 persons. (B) Cumulative cases per 100,000 persons. All curves are 7-day rolling averages. The early peak in rural cases in June 2020 was likely due to a mass testing event within one or more large businesses.

**Fig 6:**
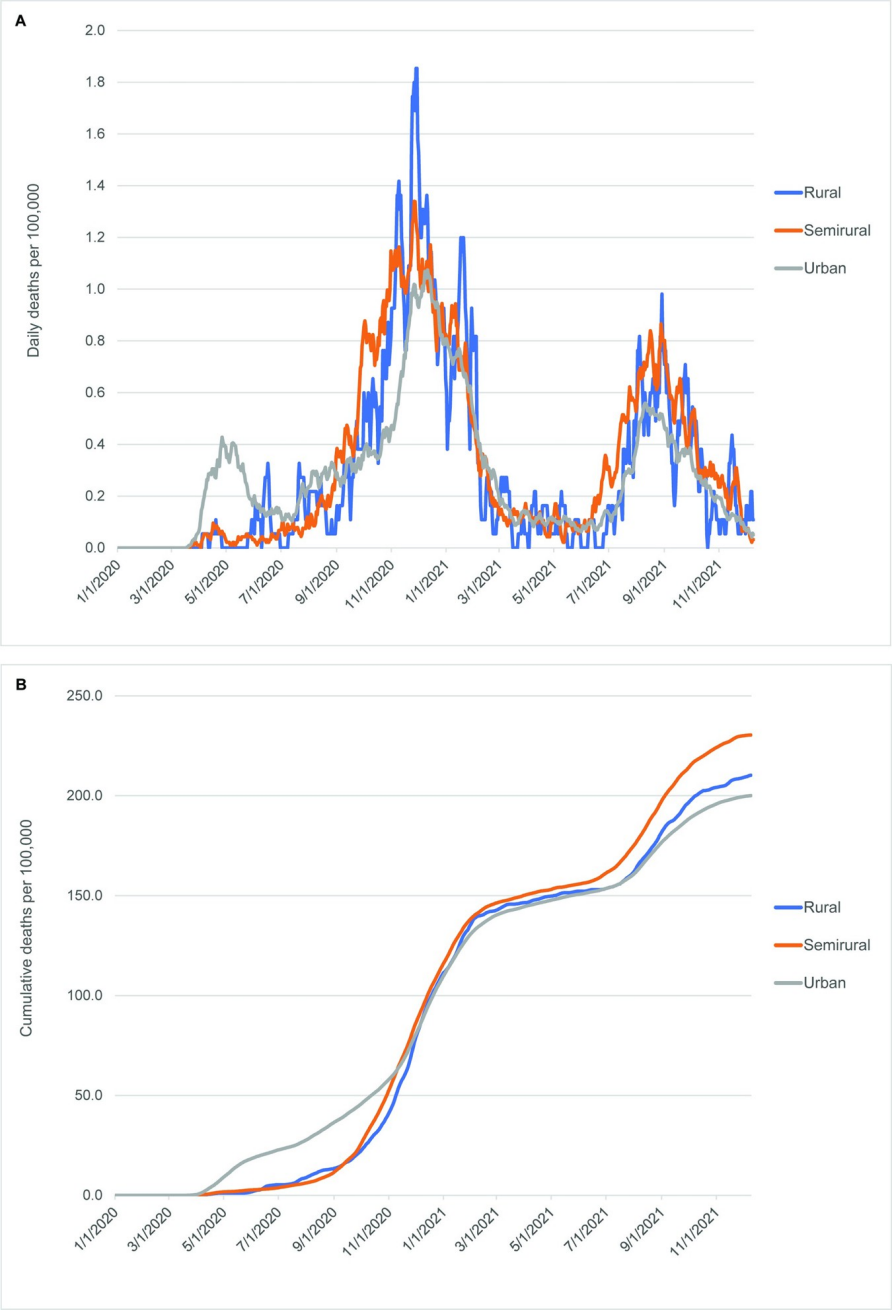
Daily and cumulative COVID-19 mortality curves among groups defined by rural status, March 2020-December 2021. (A) Daily deaths per 100,000 persons. (B) Cumulative deaths per 100,000 persons. All curves are 7-day rolling averages.

During the COVID-19 pandemic between March 2020 and December 2021, early introduction of the virus into the urban RSU group is apparent, as it shows a quicker rise in both cases and deaths (Figs 5 and 6). The higher mortality rate in the urban RSU group during the first six months of the pandemic is especially marked (Fig 6). Through most of the time period included in this study, the rural RSU group exhibited fewer cases per 100,000 persons (Fig 5A), but its cumulative mortality rates were similar to those seen in the urban RSU group from December 2020 through July 2021 (Fig 6A) before rising above the urban RSU group. Mortality rates in the semirural RSU group occurred at rates similar to the rural RSU group through June 2021, but after that they increased markedly in comparison to the urban and rural RSU groups. A comparison of cumulative case rates with cumulative mortality rates, especially between November and the end of the study period (Figs 5B and 6B), indicates that the semirural RSU group had similar case rates to the urban RSU group, but much higher mortality rates.

### Analysis by sociodemographic region

**Fig 7:**
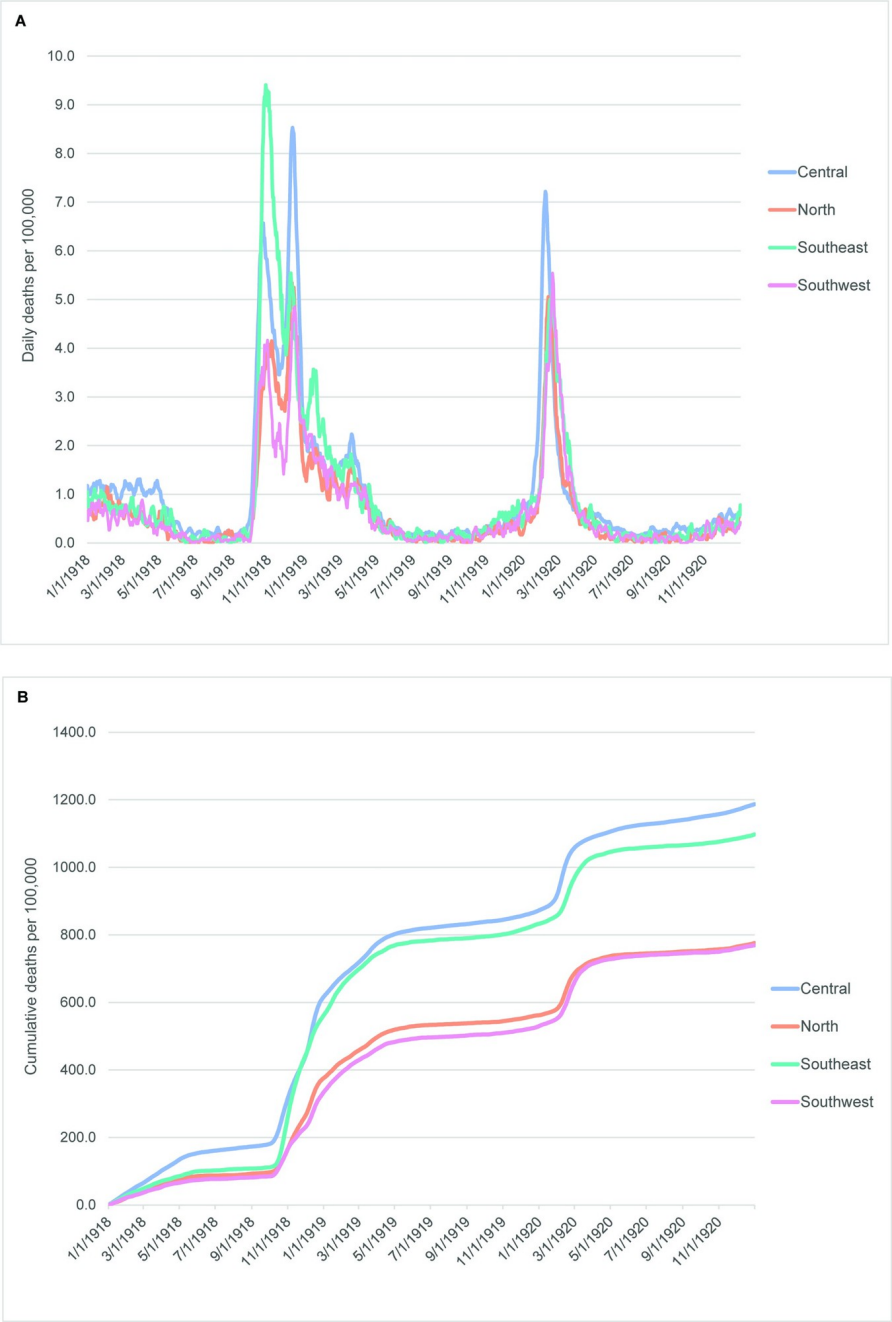
Daily and cumulative influenza mortality curves among sociodemographic clusters, 1918–1920. (A) Daily deaths per 100,000 persons. (B) Cumulative deaths per 100,000 persons. All curves are 7-day rolling averages.

The timing of the major peaks during the 1918–20 influenza pandemic was similar in all sociodemographic regions (Fig 7A), although the parallel trajectories observed in the RSU curves (cf. Fig 4) do not become apparent until after the end of the third wave in late Spring 1919 (Fig 7B). The later development of parallel trajectories may be related to a moderate peak in January 1919 observed only in the Southeast region (Fig 7A). The cumulative mortality curves separate into two distinct groups, Central/Southeast and North/Southwest (Fig 7B). The latter two regions are similar to each other throughout the pandemic; the Central and Southeast regions are distinct from one another until the rise of the second wave in October 1918. The Southeast region is aligned with the North and Southwest regions prior to this time while the Central region has substantially higher cumulative mortality than all other parts of the state. The mortality for both the Central and Southeast regions rises very quickly at the beginning of the second wave, and the two regions become similar to one another by the beginning of November 1918, although the cumulative mortality in the Central region remains higher than what was observed in the Southeast region throughout most of the pandemic.

The Central region defined for the influenza pandemic contains all the urban areas present in the state and is clearly the most densely populated region. This is likely a major reason for the higher cumulative mortality levels throughout the pandemic. This higher mortality is especially apparent during the mild first wave when the Central region was distinct from all other regions.

All regions experienced significant peaks in influenza mortality rates in 1920, with Kansas City and St. Louis experiencing the highest rates per 100,000 persons (Fig 7A). The impact of the 1920 wave is also visible in the cumulative mortality curves as a rapid rise beginning in February 1920 and a continued positive slope after that rise, especially in the Central and Southeast regions (Fig 7B).

To further examine the potential associations between sociodemographic characteristics and the severity of the influenza pandemic, the important sociodemographic variables identified using the random forest algorithm mentioned previously (average farm value, proportion White, and literacy) were compared between the two groups, Central/Southeast and North/Southwest. Uncorrected, one-sided T-tests show that the Central/Southeast clustered counties had significantly lower mean literacy rates and proportion White (p ≈ 0 for both variables). Average farm values for these combined regions are not significantly different at the .05 level (p = 0.06). See the S2 Fig, for boxplot comparisons of these variables.

**Fig 8:**
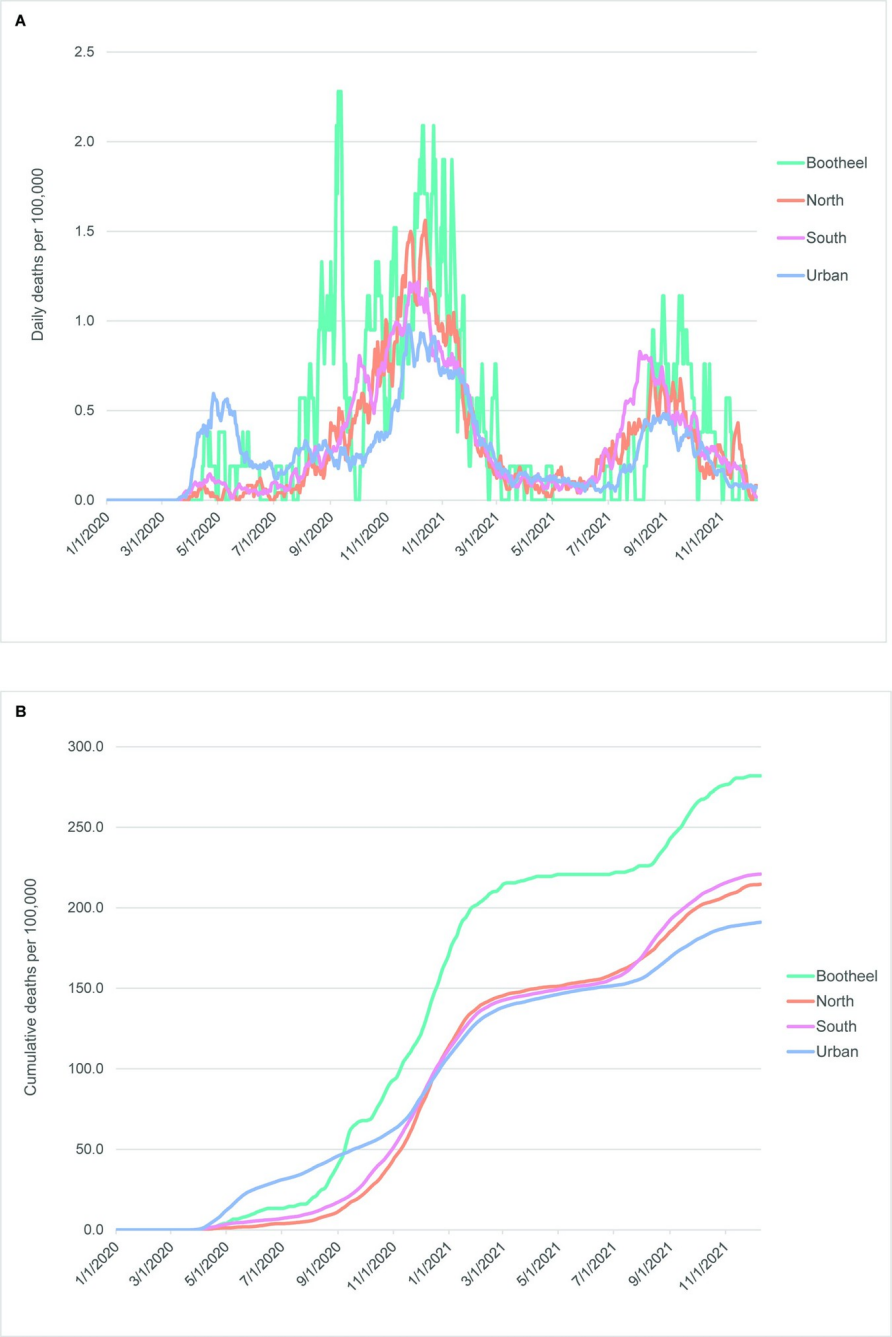
Daily and cumulative COVID-19 mortality curves among sociodemographic clusters, March 2020-December 2021. (A) Daily deaths per 100,000 persons. (B) Cumulative deaths per 100,000 persons. All curves are 7-day rolling averages.

Mortality during the 2020–21 COVID-19 pandemic shares some features with the 1918–20 influenza pandemic, including higher mortality in urban areas at the beginning of the pandemic and exceptionally high mortality in the southeastern section of the state (in this case, only in the four counties furthest to the south and east–the “Bootheel” counties) (Fig 8). The North and South counties had nearly identical mortality patterns throughout the pandemic, as was the case during the 1918–20 influenza pandemic, although in that pandemic, most counties in the southeastern quadrant were clustered together with the Bootheel counties.

**Fig 9:**
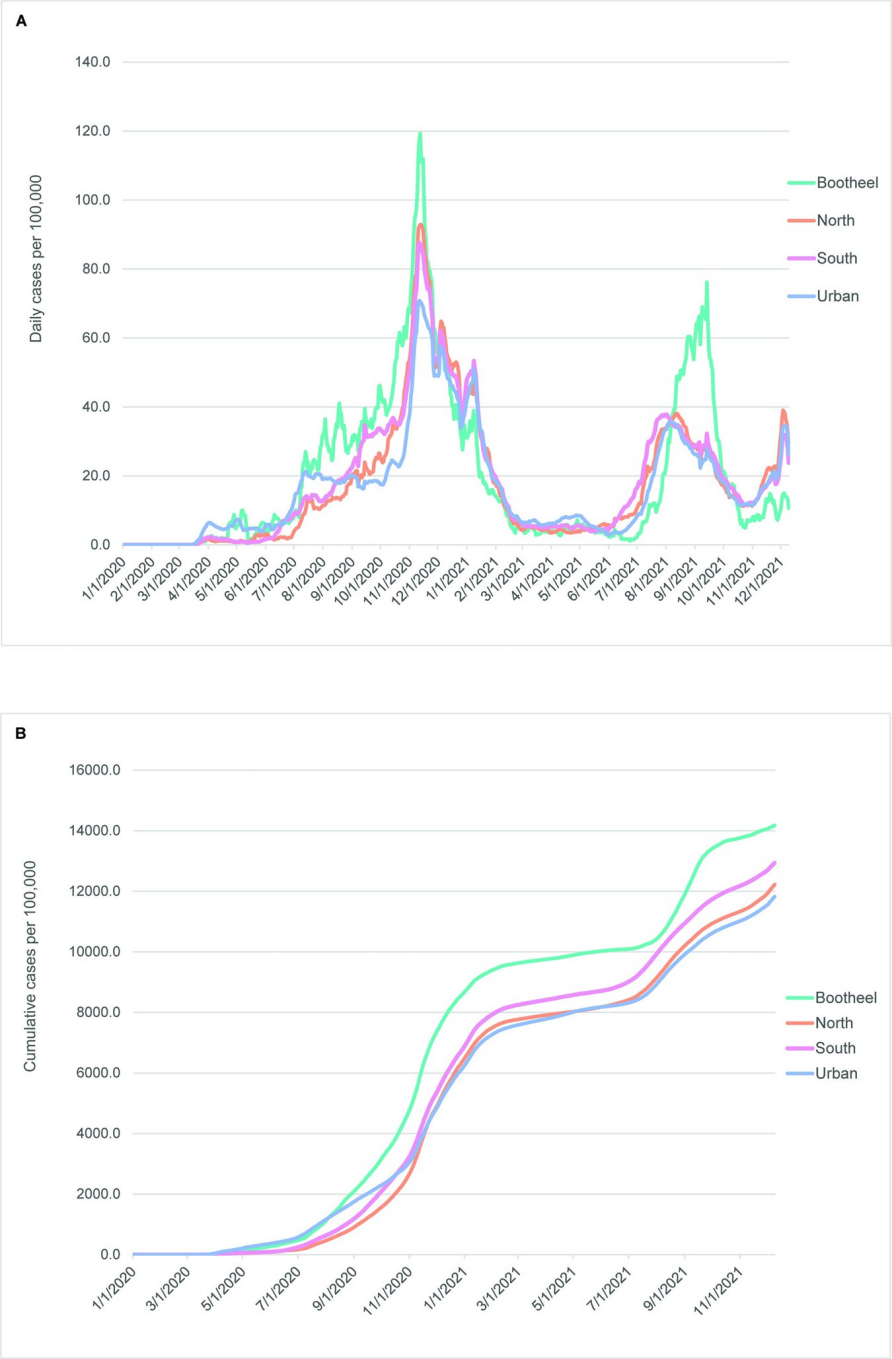
Daily and cumulative COVID-19 cases among sociodemographic clusters, March 2020-December 2021. (A) Daily cases per 100,000 persons. (B) Cumulative cases per 100,000 persons. All curves are 7-day rolling averages.

Case numbers per 100,000 population during the COVID-19 pandemic indicate that the Bootheel region was hit much harder by the pandemic than other regions when corrected for differences in population size. As Fig 9 shows, both the daily incidence curves and the cumulative case rates in this region were substantially higher than observed in all other regions throughout much of the time period included in this study.

To further examine the potential associations between sociodemographic characteristics and the severity of the 2020–21 COVID-19 pandemic in the Bootheel, the four counties in that region were aggregated and compared to the combined North, South, and Urban counties. Mean comparisons were conducted using simple, one-sided T-tests on these two sets of counties. The Bootheel-clustered counties have significantly lower literacy levels (p ≈ 0) and proportion White (p = 0.01) than the rest of the state. The average farm value for Bootheel counties is significantly higher (p = 0.001). The proportion of Hispanics and age ratios are not significantly different. It should be noted that the Bootheel cluster includes only a small number of counties (4 out of 115 total Missouri counties) which lends less credibility to these mean comparisons due to possible undetected issues with distributional assumptions required for the statistical tests. However, as the small sample does not appear to be heavily skewed (see S3 Fig), there is still reasonable evidence to support the objective validity of these conclusions.

The analyses of data from both pandemics indicates that the southeastern part of the state experienced more severe pandemic outcomes during both pandemics, but this impact appears to have become more localized to the Bootheel region rather than the entire southeast in the 21^st^ century. As discussed above, the most important explanatory variables, literacy, average farm value, and proportion White, were the same in both time periods, supporting the conclusion that negative outcomes during pandemics may result from long-standing disparities in education and factors, such as generational poverty and limited access to resources, that may vary differentially among racial and ethnic groups.

## Discussion

Numerous factors influence the patterns of spread and severity of a pandemic at any given time or when looking at two pandemics in the same place at different times, such as the 1918–20 influenza and the 2020–23 COVID-19 pandemics. These factors include, for example, biological differences and/or evolutionary shifts in the pathogens, environmental differences such as changes in climate or settlement patterns, the underlying health and nutritional status of the populations involved. A full discussion of these factors is beyond of the scope of this paper; therefore, we limit our discussion here to similarities and differences in the two pandemics under discussion, the base populations through which they were spreading, and changes in the sociodemographic characteristics for which we have found data from both the early 20^th^ and early 21^st^ centuries.

There were more differences between the Missouri of 1918 and the Missouri of 2020 than there were similarities, but the similarities were important. All counties classified as urban in 1918 are also classified as urban in 2020 and every county that is fully rural today was also fully rural in 1918. The primary region that saw a major shift was the area comprised of counties in the center of the state, most of which were small and rural in 1918, but which now have a clearly defined urban core that ranks as the 3^rd^ most populated region behind the greater Kansas City and St. Louis areas.

A second, and somewhat unexpected similarity between 1918 and 2020 is that the sociodemographic variables that explained the greatest amount of variation among the state’s counties were the same for the two time periods and were ranked in the same order. These included the average farm value, the proportion of the population that was White, and the level of literacy, in that order. For the 2020–21 COVID-19 pandemic only, the proportion Hispanic and the age distribution are of measurable but lesser importance. The similarity in significant variables may be partly a function of the small number of variables and the constraints that they were not strongly correlated with each other and were available from both time periods.

Several major changes in the population of Missouri since 1918 likely had significant effects on infectious disease patterns. One of the most important is that most counties designated as rural or semirural now have substantial urban concentrations, even though, by definition, over half the counties’ populations live in rural areas. Since the transmission of contagious pathogens is facilitated by higher population densities, this clustering into larger towns and cities may have increased the opportunity for COVID-19 transmission in comparison to influenza transmission in 1918. Counties are also more connected to one another and the rates of travel, including for commuting among and between counties, are presumed to be much higher than they used to be. In contrast, for the 1918–20 influenza pandemic, once the pandemic took hold within a county, there appears to have been little difference in transmission risk based on the RSU status of a county, as evidenced by the parallel increases in cumulative mortality and assuming that mortality rates are closely tied to infection rates at the population level. Also, it is important to note that even though the five cities (St. Louis City, Kansas City, St. Joseph, Springfield, and Joplin) were scattered across the state, little measurable difference in timing of the mortality peaks among these places was observed, suggesting that influenza did not spread across regions slowly, but perhaps was introduced multiple times in multiple cities in a short period of time.

One major change in the structure of life between 1918 and 2020 is a shift from a farm-based economy to one that is service-based. Less of the population is involved in relatively solitary agricultural work, so the opportunities for work-related pathogen transmissions are probably substantially higher now. Thus, the primary pathways for transmission are likely to be different now from what they were 100 years ago.

The nature of access to health care and the resources available perhaps resulted in the most marked changes between 1918 and 2020. Although the number of physicians per capita was higher in urban than in rural centers even in 1918, physicians during that time were predominantly general practitioners rather than the specialists of the present time. When the general similarity in medical training of the majority of physicians is considered in conjunction with a lack of effective treatments for influenza in 1918, this likely means that the outcomes of severe cases of influenza would be relatively homogeneous across the population. In other words, two people in different parts of the state with equally severe cases would be more likely to have similar outcomes (e.g., severe long-term effects or death) than might be the case today with greater availability of specialized treatments and heterogenous access to health care across population groups.

Related to this is the emphasis now on paying for medical care with health insurance. This was not a factor in 1918, but has had strong impacts on health in the 21^st^ century. Rural populations in Missouri have much lower rates of insurance coverage [73], as do rural populations throughout the U.S. In a study of factors influencing mortality rates from COVID-19, Grekousis et al. [74] noted that in most of Missouri’s counties, the percentage of the population with no insurance was the most important factor explaining COVID-19 mortality rates.

How do the 1918–20 influenza and 2020–21 COVID-19 pandemics in Missouri compare to each other? One marked similarity between the two pandemics is the presence of distinct epidemic waves. However, the timing of these waves differs. The 1918–20 influenza pandemic was characterized by mostly winter-spring waves in 1918, 1919, and 1920. The 2020–21 COVID-19 pandemic appears to have biannual waves, with one occurring in the fall/winter and a second in the summer. The method used to estimate the excess mortality during each pandemic does take these basic differences into account, as it uses the normal seasonally varying patterns of a region during non-epidemic times to generate a reasonable baseline expected prevalence.

Another similarity between the pandemics is that in both outbreaks, the severity, as measured by number of deaths per 100,000 persons during the influenza pandemic and number of cases per 100,000 persons during the 2020–21 COVID-19 pandemic, was lower in rural counties than in semirural counties, which was lower than in urban counties. Interestingly, the number of deaths per 100,000 persons during the COVID-19 pandemic showed the same relationship as the influenza pandemic from its beginning March 2020 until November 2020, but subsequently the mortality rates in semirural counties were markedly higher than those in urban counties and the mortality rates in rural counties were somewhat higher than those in urban counties. Matthews et al. [14] and Ullrich and Mueller [75] observed a similar crossover in county-level data from the entire U.S. The difference between the two pandemics may reflect higher population densities and lower levels of social isolation in semirural counties in comparison to rural areas, facilitating spread of the pandemic, but a magnification of the risk of poorer outcomes after infection because of greater heterogeneity in the distribution of health care resources, leading to delays in and difficulties accessing adequate health care outside the more urbanized regions.

Our data and analyses clearly indicate that the Bootheel region of Missouri has been especially affected by the 2020–21 COVID-19 pandemic (Fig 7). As Fig 1B illustrates, this region is sociodemographically distinct from the rest of the state. According to the Missouri Economic Research and Information Center, the southeast region of the state, which includes the Bootheel, has an aging workforce, a higher percentage of residents with a disability, and a lower percentage of residents with post-secondary degrees compared to the rest of the state [35]. The Missouri Department of Health and Senior Services reported that all of the Bootheel counties have persistent overall poverty [73].

It is important to recognize limitations of this study. Perhaps the most important one is that the set of sociodemographic variables chosen for the study does not include many variables that likely affected disease patterns in one or both pandemics. An important variable that was not included in analyses of either the 1918–20 Influenza or the 2020–21 COVID-19 data was age (other than the crude ratio “population aged 25-44/population aged 65+”), primarily because sufficient data to estimate populations at risk by age were not available at the county level in 1918. Above the county level, outside the large metropolitan areas age distributions were only available for the statewide rural population. To maintain comparability between the two time periods, we focused our analyses on crude mortality rates since we did not have data for age-adjustment in the 1910 period. We also limited our data set to a small number of variables that captured at least some aspects of the significant dimensions (e.g., population structure, education, health care) that would be important in determining patterns of spread of infectious diseases.

Another limitation is the scheme used to categorize each county as rural, semirural or urban. Several studies related to the 2020–21 COVID-19 pandemic have used the 2013 NCHS urban-rural classification scheme [76], which applies a micropolitan-metropolitan set of criteria to categorize each county in the U.S. This classification usually considers entire counties and the access the county population has to urban centers rather than the density of people living in a local area [77]. Population density is perhaps the most important issue to consider when thinking about infectious disease transmission, however, and for respiratory pathogens such as influenza and SARS-CoV-2, such transmission can only occur when people come into direct and close contact with one another. Population density is a better measure of the risk of close contact than access to the resources of urban centers and this factor is captured better by using the U.S. Census designations of urban and rural rather than the metropolitan/micropolitan scheme.

The lack of availability of case data from 1918–20 is an additional limitation. This constrains the study to direct comparisons of mortality only during the two pandemics. In 1918 there were no effective treatments for influenza and although there were undoubtedly differences in quality of supportive care in different regions due to both socioeconomic issues and individual personalities, it is reasonable to assume that the likelihood of severe outcomes among those infected varied much less than is the case with the present COVID-19 pandemic. Given this, we have assumed that the 1918–20 influenza mortality patterns would closely approximate morbidity patterns from that time, albeit with a time lag that relates to the average time from infection to death. We are not able to make this assumption for the 2020–21 COVID-19 pandemic, as it has been clear since the beginning of the pandemic that the risk of mortality varies markedly among socioeconomic classes and across different regions. Our comparisons of the 1918–20 influenza pandemic and the 2020–21 COVID-19 pandemic must be tempered by careful consideration of this issue.

The results of this study have important implications for understanding and predicting the impact of future epidemics. In particular, they emphasize the continuing and significant difficulties faced by impoverished areas of Missouri, such as the Bootheel. In spite of the general quality of care available in the U.S. today, these regions still experience differential and negative health consequences in the face of infectious disease epidemics. Orford et al. [30] observed a similar persistence of poverty and its negative impacts on health from the late 19th century in London to the end of the 20^th^ century and concluded that spatial patterns of poverty are extremely robust and difficult to change. Their and our studies suggest that solutions to health issues facing impoverished people will benefit not only from creative approaches but also from an understanding of the historical factors that have led to and continue to maintain those impoverished situations.

Our results show that, contrary to what one might expect, areas that are neither fully rural nor urban experienced the worst outcomes during the 2020–21 COVID-19 pandemic in Missouri. Most likely this is also a resource-related issue–people who live in such areas have urban-like contact patterns that increase the chance of disease transmission, but many of these county residents also may have diminishing resources to secure high quality and easily accessible health care in the face of disease. Without dealing with both long- and short-term practices affecting the differential distribution of resources and universal high-quality health care, the economically disadvantaged and those living in semirural areas of the U.S. are destined to remain differentially affected during pandemics.

## Supporting information

S1 Table Classification of Missouri counties by rurality and sociodemographic region. (PDF)

S1 File Sociodemographic and 1918–20 influenza pandemic data used in the study. (XLSX)

S2 File Monthly all-cause death counts, estimated population, and excess mortality estimates. (XLSX)

S1 Fig Pandemic deaths in Missouri during the 1918 influenza and 2020 COVID-19 pandemics. (A) Pneumonia and influenza deaths from 1/1/1918 to 12/13/1920. (B) COVID-19 deaths from 1/1/2020 to 3/9/2022.
(PDF)

S2 Fig Comparisons of sociodemographic variables in 1910, Central/Southeast vs. North/Southwest. (PDF)

S3 Fig Comparisons of sociodemographic variables in 2020, the Bootheel vs. the rest of Missouri. (PDF)
