# Supplementary material for: Associations between rurality and regional differences in sociodemographic factors and the 1918–20 influenza and 2020–21 COVID-19 pandemics in Missouri counties: An ecological study
Source: PLoS One. 2023 Aug 30;18(8):e0290294. doi: 10.1371/journal.pone.0290294 (PMC10468050; doi:10.1371/journal.pone.0290294)
Supplement: S1 Table — (PDF) [file pone.0290294.s001.pdf]

Table S1. Classification of Missouri counties by rural/urban status (RSU) and sociodemographic region, 1910 and 2010.*

	1910			*2010*			
**County**	**% rural**	**RSU^ Class**	**Region**	**% rural**	**RSU^ Class**	**Region**	**RSU^ change**
Adair	72	semirural	North	38	urban	North	S → U
Andrew	100	rural	North	61	semirural	North	R → S
Atchison	100	rural	North	100	rural	North	
Audrain	73	semirural	Central	41	urban	North	S → U
Barry	83	semirural	Southwest	73	semirural	South	
Barton	100	rural	Southwest	64	semirural	South	R → S
Bates	78	semirural	North	77	semirural	South	
Benton	100	rural	Southwest	87	semirural	South	R → S
Bollinger	100	rural	Southeast	100	rural	South	
Boone	68	semirural	Central	19	urban	Urban	S → U
Buchanan	17	urban	Central	13	urban	Urban	
Butler	67	semirural	Southeast	52	semirural	South	
Caldwell	100	rural	North	100	rural	North	
Callaway	79	semirural	Central	62	semirural	North	
Camden	100	rural	Southwest	74	semirural	South	R → S
Cape Girardeau	69	semirural	Southeast	31	urban	South	S → U
Carroll	85	semirural	North	65	semirural	North	
Carter	100	rural	Southeast	100	rural	South	
Cass	100	rural	North	32	urban	North	R → U
Cedar	84	semirural	Southwest	75	semirural	South	
Chariton	100	rural	Central	100	rural	North	
Christian	100	rural	Southwest	45	urban	South	R → U
Clark	100	rural	North	100	rural	North	
Clay	66	semirural	North	10	urban	Urban	S → U
Clinton	81	semirural	North	76	semirural	North	
Cole	46	urban	Central	29	urban	South	
Cooper	79	semirural	Central	53	semirural	North	
Crawford	100	rural	Southeast	73	semirural	South	R → S
Dade	100	rural	Southwest	100	rural	South	
Dallas	100	rural	Southwest	82	semirural	South	R → S
Daviess	100	rural	North	100	rural	North	
Dekalb	100	rural	North	62	semirural	Urban	R → S
Dent	100	rural	Southeast	69	semirural	South	R → S
Douglas	100	rural	Southeast	79	semirural	South	R → S
Dunklin	90	semirural	Southeast	51	semirural	Bootheel	
Franklin	88	semirural	Central	56	semirural	South	
Gasconade	100	rural	Central	81	semirural	North	R → S
Gentry	100	rural	North	100	rural	North	

Greene	45	urban	Central	14	urban	South	
Grundy	66	semirural	North	46	urban	North	S → U
Harrison	100	rural	North	70	semirural	North	R → S
Henry	82	semirural	North	50	urban	South	S → U
Hickory	100	rural	Southwest	100	rural	South	
Holt	100	rural	North	100	rural	North	
Howard	84	semirural	Central	64	semirural	North	
Howell	86	semirural	Southeast	72	semirural	South	
Iron	100	rural	Southeast	75	semirural	South	R → S
Jackson	9	urban	Central	4	urban	Urban	
Jasper	35	urban	Central	24	urban	South	
Jefferson	74	semirural	Southeast	30	urban	South	S → U
Johnson	82	semirural	North	50	semirural	Urban	
Knox	100	rural	North	100	rural	North	
Laclede	100	rural	Southwest	61	semirural	South	R → S
Lafayette	74	semirural	Central	57	semirural	North	
Lawrence	84	semirural	Southwest	59	semirural	South	
Lewis	100	rural	North	100	rural	North	
Lincoln	100	rural	Central	75	semirural	South	R → S
Linn	62	semirural	North	67	semirural	North	
Livingston	68	semirural	North	37	urban	North	S → U
McDonald	100	rural	Southwest	100	rural	South	
Macon	88	semirural	North	68	semirural	North	
Madison	77	semirural	Southeast	66	semirural	South	
Maries	100	rural	Southwest	100	rural	North	
Marion	40	urban	Central	25	urban	North	
Mercer	100	rural	North	100	rural	North	
Miller	100	rural	Southwest	80	semirural	North	R → S
Mississippi	78	semirural	Southeast	33	urban	Bootheel	S → U
Moniteau	100	rural	Southwest	53	semirural	North	R → S
Monroe	100	rural	Central	100	rural	North	
Montgomery	100	rural	Central	79	semirural	North	R → S
Morgan	100	rural	Southwest	100	rural	South	
New Madrid	100	rural	Southeast	57	semirural	Bootheel	R → S
Newton	87	semirural	Southwest	64	semirural	South	
Nodaway	84	semirural	North	43	urban	Urban	S → U
Oregon	100	rural	Southeast	81	semirural	South	R → S
Osage	100	rural	Central	100	rural	North	
Ozark	100	rural	Southeast	100	rural	South	
Pemiscot	81	semirural	Southeast	49	urban	Bootheel	S → U
Perry	100	rural	Southeast	56	semirural	South	R → S
Pettis	47	urban	Central	38	urban	Urban	

Phelps	100	rural	Southwest	46	urban	South	R → U
Pike	80	semirural	Central	54	semirural	North	
Platte	100	rural	North	16	urban	Urban	R → U
Polk	100	rural	Southwest	69	semirural	South	R → S
Pulaski	100	rural	Southwest	44	urban	Urban	R → U
Putnam	100	rural	North	100	rural	North	
Ralls	100	rural	Central	96	semirural	North	R → S
Randolph	58	semirural	Central	45	urban	North	S → U
Ray	83	semirural	North	75	semirural	North	
Reynolds	100	rural	Southeast	100	rural	South	
Ripley	100	rural	Southeast	100	rural	South	
St. Charles	62	semirural	Central	6	urban	South	S → U
St. Clair	100	rural	Southwest	100	rural	South	
Ste. Genevieve	100	rural	Southeast	76	semirural	South	R → S
St. Francois	78	semirural	Southeast	40	urban	South	S → U
St. Louis County	71	semirural	Central	1	urban	Urban	S → U
Saline	73	semirural	Central	46	urban	Urban	S → U
Schuyler	100	rural	North	100	rural	North	
Scotland	100	rural	North	100	rural	North	
Scott	85	semirural	Southeast	42	urban	South	S → U
Shannon	100	rural	Southeast	100	rural	South	
Shelby	100	rural	North	100	rural	North	
Stoddard	100	rural	Southeast	70	semirural	South	R → S
Stone	100	rural	Southeast	89	semirural	South	R → S
Sullivan	100	rural	North	100	rural	Urban	
Taney	100	rural	Southwest	44	urban	South	R → U
Texas	100	rural	Southwest	99	semirural	South	R → S
Vernon	75	semirural	Southwest	58	semirural	South	
Warren	100	rural	Central	63	semirural	North	R → S
Washington	100	rural	Southeast	81	semirural	South	R → S
Wayne	100	rural	Southeast	100	rural	South	
Webster	100	rural	Southwest	74	semirural	South	R → S
Worth	100	rural	North	100	rural	North	
Wright	100	rural	Southwest	77	semirural	South	R → S
St. Louis City	0	urban	Central	0	urban	Urban	

* Sources: For 1910 data — U.S. Census Bureau. Statistics for Missouri. Thirteenth Census of the United States Taken in the Year 1910. Washington D.C.: Government Printing Office; 1913. For 2010 data — U.S. Census Bureau. Missouri: 2010. Population and Housing Unit Counts. 2010 Census of Population and Housing; 2012; https://www2.census.gov/library/publications/decennial/2010/cph-2/cph-2-27.pdf.

^ R — rural; S — semirural; U — urban
