# Supplementary figures and images for: Associations between rurality and regional differences in sociodemographic factors and the 1918–20 influenza and 2020–21 COVID-19 pandemics in Missouri counties: An ecological study
Source: PLoS One. 2023 Aug 30;18(8):e0290294. doi: 10.1371/journal.pone.0290294 (PMC10468050; doi:10.1371/journal.pone.0290294)

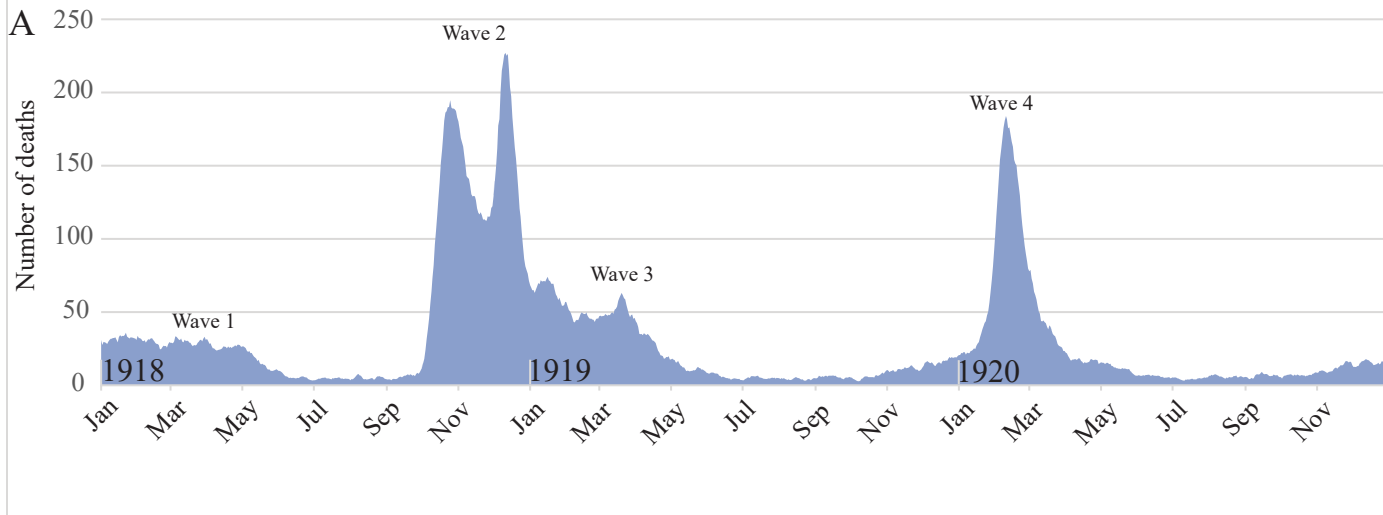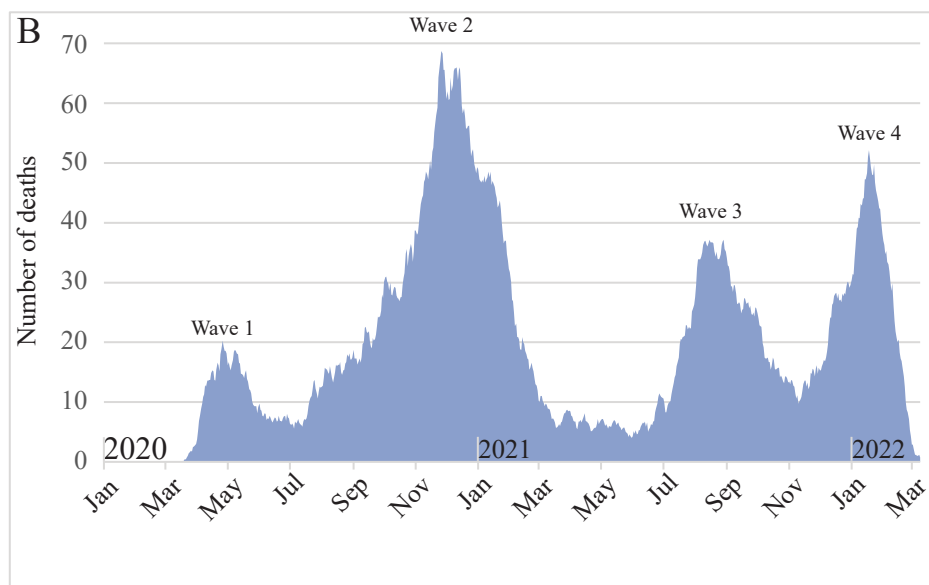

Supplement: S1 Fig — (A) Pneumonia and influenza deaths from 1/1/1918 to 12/13/1920. (B) COVID-19 deaths from 1/1/2020 to 3/9/2022. (PDF) [file pone.0290294.s004.pdf]

Ave Farm Value

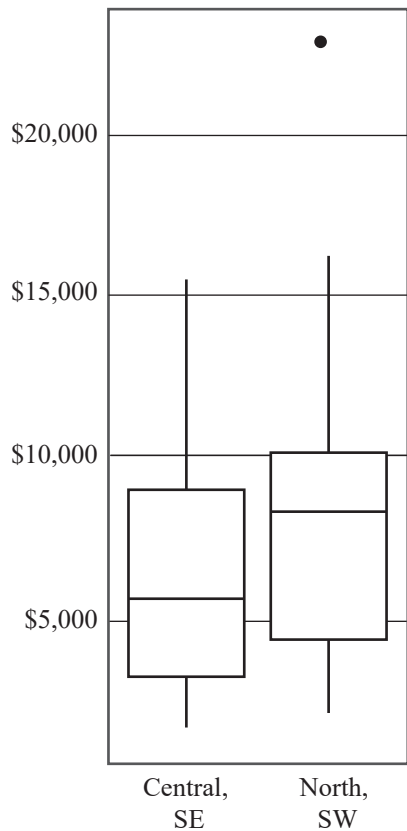

Proportion White

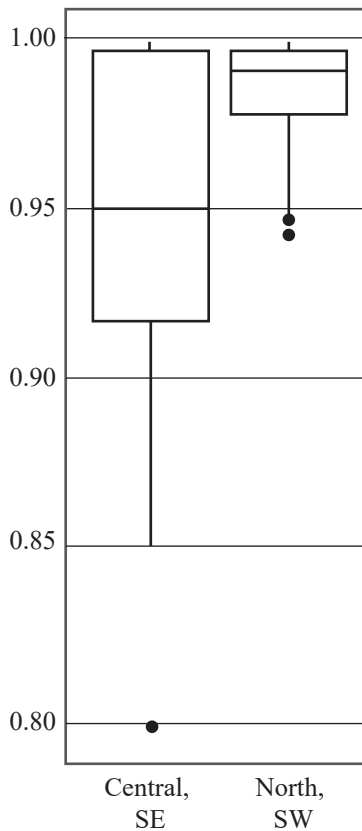

Pct Literacy

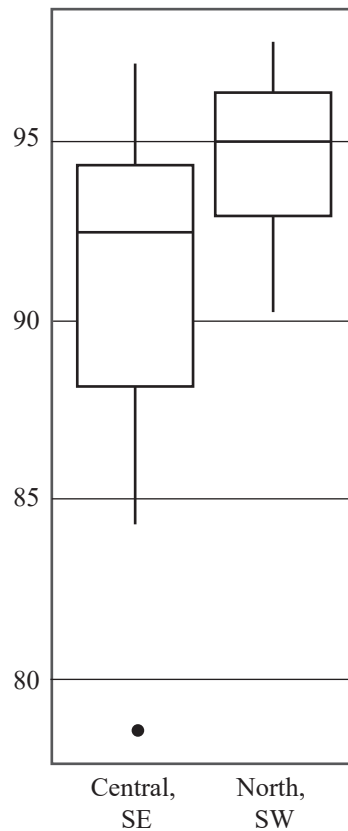

Supplement: S2 Fig — (PDF) [file pone.0290294.s005.pdf]

Ave Farm Value

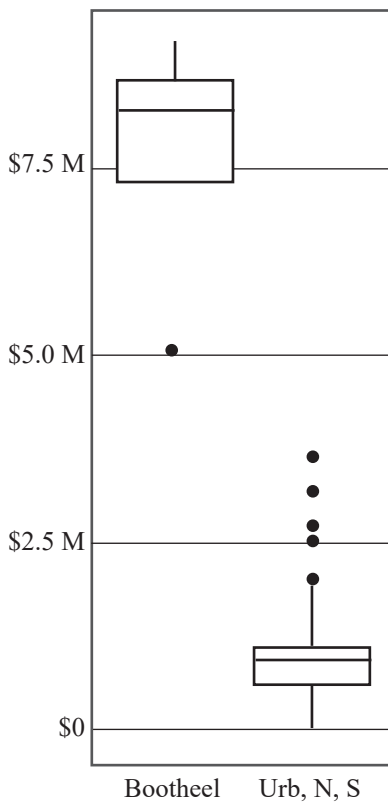

Proportion White

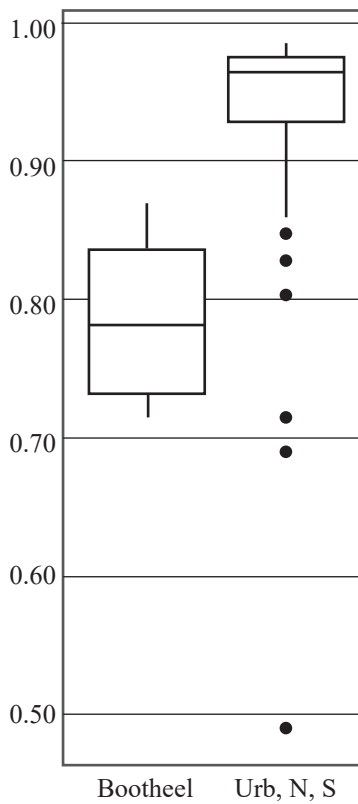

Pct Literacy

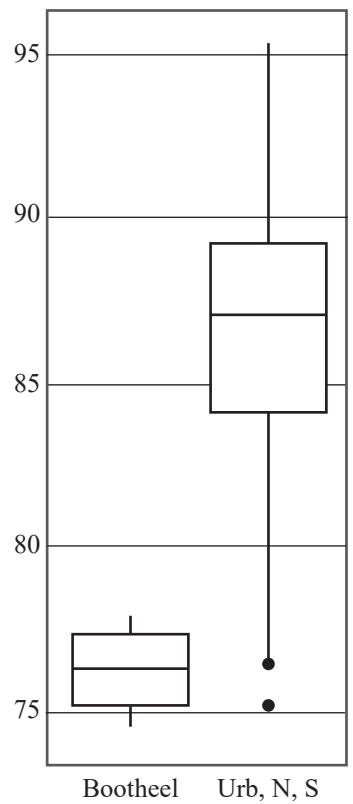

Proportion Hispanic

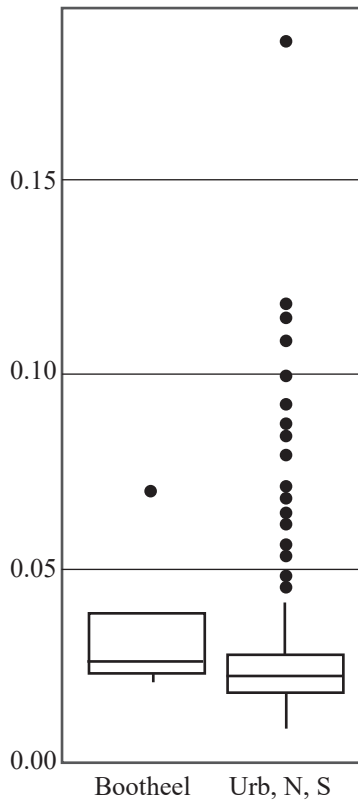

Young/Old Ratio

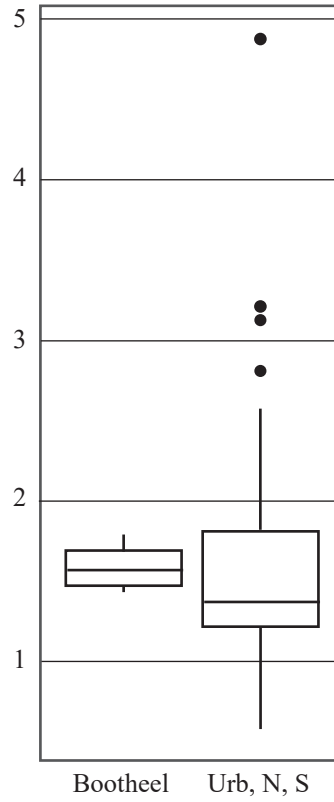

Supplement: S3 Fig — (PDF) [file pone.0290294.s006.pdf]
